# TEST (Trial of Eczema allergy Screening Tests): protocol for feasibility randomised controlled trial of allergy tests in children with eczema, including economic scoping and nested qualitative study

**DOI:** 10.1136/bmjopen-2018-028428

**Published:** 2019-05-09

**Authors:** Matthew J Ridd, Louisa Edwards, Miriam Santer, Joanne R Chalmers, Lisa Waddell, Deborah Marriage, Ingrid Muller, Kirsty Roberts, Kirsty Garfield, Joanna Coast, Lucy Selman, Clare Clement, Alison R G Shaw, Elizabeth Angier, Peter S Blair, Nicholas L Turner, Jodi Taylor, Joe Kai, Robert J Boyle

**Affiliations:** 1 Population Health, University of Bristol, Bristol, UK; 2 Faculty of Health Sciences, Simon Fraser University, Burnaby, British Columbia, Canada; 3 Primary Care and Population Sciences, University of Southampton, Southampton, UK; 4 Centre for Evidence Based Dermatology, University of Nottingham, Nottingham, UK; 5 Nottingham CityCare, Nottingham, UK; 6 Bristol Royal Hospital for Children, Bristol, UK; 7 Bristol Randomised Trials Collaboration, University of Bristol, Bristol, UK; 8 Health Economics at Bristol, University of Bristol, Bristol, UK; 9 Centre for Academic Child Health, University of Bristol, Bristol, UK; 10 Division of Primary Care, University of Nottingham, Nottingham, UK; 11 Imperial College London, London, UK

**Keywords:** eczema, allergy, clinical trials

## Abstract

**Background:**

Early onset eczema is associated with food allergy, and allergic reactions to foods can cause acute exacerbations of eczema. Parents often pursue dietary restrictions as a way of managing eczema and seek allergy testing for their children to guide dietary management. However, it is unclear whether test-guided dietary management improves eczema symptoms, and whether the practice causes harm through reduced use of conventional eczema treatment or unnecessary dietary restrictions. The aim of the Trial of Eczema allergy Screening Tests Study is to determine the feasibility of conducting a trial comparing food allergy testing and dietary advice versus usual care, for the management of eczema in children.

**Methods and analysis:**

Design: A single centre, two-group, individually randomised, feasibility randomised controlled trial (RCT) with economic scoping and a nested qualitative study. Setting: General Practioner (GP) surgeries in the west of England. Participants: children aged over 3 months and less than 5 years with mild to severe eczema. Interventions: allergy testing (structured allergy history and skin prick tests) or usual care. Sample size and outcome measures: we aim to recruit 80 participants and follow them up using 4-weekly questionnaires for 24 weeks. Nested qualitative study: We will conduct ~20 interviews with parents of participating children, 5–8 interviews with parents who decline or withdraw from the trial and ~10 interviews with participating GPs. Economic scoping: We will gather data on key costs and outcomes to assess the feasibility of carrying out a cost-effectiveness analysis in a future definitive trial.

**Ethics and dissemination:**

The study has been reviewed by the Health Research Authority and given a favourable opinion by the NHS REC (West Midlands – South Birmingham Research Ethics Committee, Reference Number 18/WM/0124). Findings will be submitted for presentation at conferences and written up for publication in peer-reviewed journals, which may include mixed-method triangulation and integration of the quantitative and qualitative findings.

**Trial registration:**

ISRCTN15397185; Pre-results.

**Strength and limitations**

- This is the first randomised controlled trial exploring test-guided dietary management for treating eczema to be done in a primary care setting, where most children with eczema are diagnosed and managed in the UK.
- Data on the processes and outcomes that are being collected will help determine the feasibility of a definitive trial and associated economic evaluation.
- The nested qualitative study will help to interpret and explain the quantitative feasibility findings and to generate new knowledge around the issues of food allergy, allergy tests and dietary modification in children with eczema, from the perspective of parents and GPs.
- The study is being conducted in a single centre in the west of England, which may limit the generalisability of the findings.

## Introduction

### Background and rationale

Childhood eczema is a common long-term condition characterised by dry and itchy skin. In accordance with the recommended nomenclature of the World Allergy Organisation, we use the label ‘eczema’ to refer to the clinical phenotype of atopic eczema/dermatitis.^1^

Eczema affects around 20% of preschool age children; 60% of these develop symptoms in the first year of life and 90% by 5 years of age.^2^ In the UK most children with eczema are diagnosed and managed in primary care with a combination of emollients and topical corticosteroids. Having eczema can significantly impact the quality of life of the affected child and their family. Treatment adherence can be problematic for numerous reasons, including parents/carers (hereafter, ‘parents’) seeking a ‘cure’ through dietary exclusions for possible food allergy rather than ‘control’ through long-term use of topical treatments.^3–5^

Eczema is associated with food allergy, especially early onset, troublesome eczema,^6^ and parents of children with eczema often try dietary exclusions in an attempt to reduce symptom severity and may seek allergy testing to guide such dietary exclusions. Allergic reactions to food can cause an acute exacerbation of eczema, either as part of an IgE-mediated reaction or as an isolated non-IgE mediated reaction to a food (see box 1). Parents’ suspicions of food allergies in general and especially with respect to eczema have low specificity. Depending on the specific population studied and the definitions used, 15%–36% of children with eczema compared with about 6% of the general population have a food sensitivity (a ‘positive’ test result, without clinical symptoms) or allergy.^7^ Clinical practice in offering allergy tests to parents of children with eczema varies significantly, with many allergy clinics routinely ‘screening’ for associated food allergies, but few primary care services offering testing in the absence of a history suggesting an IgE-mediated reaction to a food.

Box 1 **IgE-mediated and non-IgE-mediated food allergy**

- The World Allergy Organisation defines food allergy as an immune-mediated hypersensitivity reaction to food and may be divided into IgE-mediated and non-IgE-mediated reactions.^1^
- IgE-mediated food allergy involves immediate hypersensitivity (typically within 5–30 min of ingestion and always within 2 hours) through the action of mast cells. It can be reliably diagnosed when there is a typical history of reaction within 1–2 hours of exposure and demonstration of specific IgE to the relevant food on blood or skin prick testing.
- Non-IgE-mediated food allergy is delayed (between 2–48 hours postingestion) and thought to be caused by an aberrant T cell response. It is more difficult to diagnose as there are no reliable diagnostic tests other than dietary exclusions and re-introduction.^47^

A Cochrane review^8^ of dietary exclusions for adults and children with eczema published in 2008 did not find any evidence of benefit for exclusion diets in unselected populations (ie, those without clinically suspected food allergies), but did identify one trial which suggested that infants with suspected egg allergy who have positive specific IgE to eggs may benefit from an egg-free diet.^9^ While this suggests that test-guided dietary management may be worthwhile, both this and two other subsequently published systematic reviews^10 11^ have called for better-designed and conducted trials. We have not identified any economic evaluations in this area and while concerns about food allergy have been raised during in-depth interviews of parents’ general experiences of looking after children with eczema,^3–5^ and have arisen as an important concern for parents in online discussion forums,^12^ we are not aware of any qualitative work specifically exploring this issue.

### Aim and objectives

The aim of the study to determine the feasibility of conducting a trial comparing test-guided dietary management versus usual care, for the management of eczema in children.

The objectives are to explore the following factors that will determine the feasibility and inform the design of a future, full-scale clinical and cost-effectiveness randomised controlled trial (RCT):

- Participant recruitment (including numbers potentially eligible), retention and adherence to allocation/dietary advice.
- Outcome completion rates.
- Logistics of trial processes and their acceptability to participants.

### Trial design

The Trial of Eczema allergy Screening Tests (TEST) is a single-centre, two-group, individually randomised, feasibility RCT^13^ with economic scoping and nested qualitative study.

## Methods and analysis

### Study setting

Primary care (GP surgeries) in the west of England.

### Recruitment

**Figure 1:**
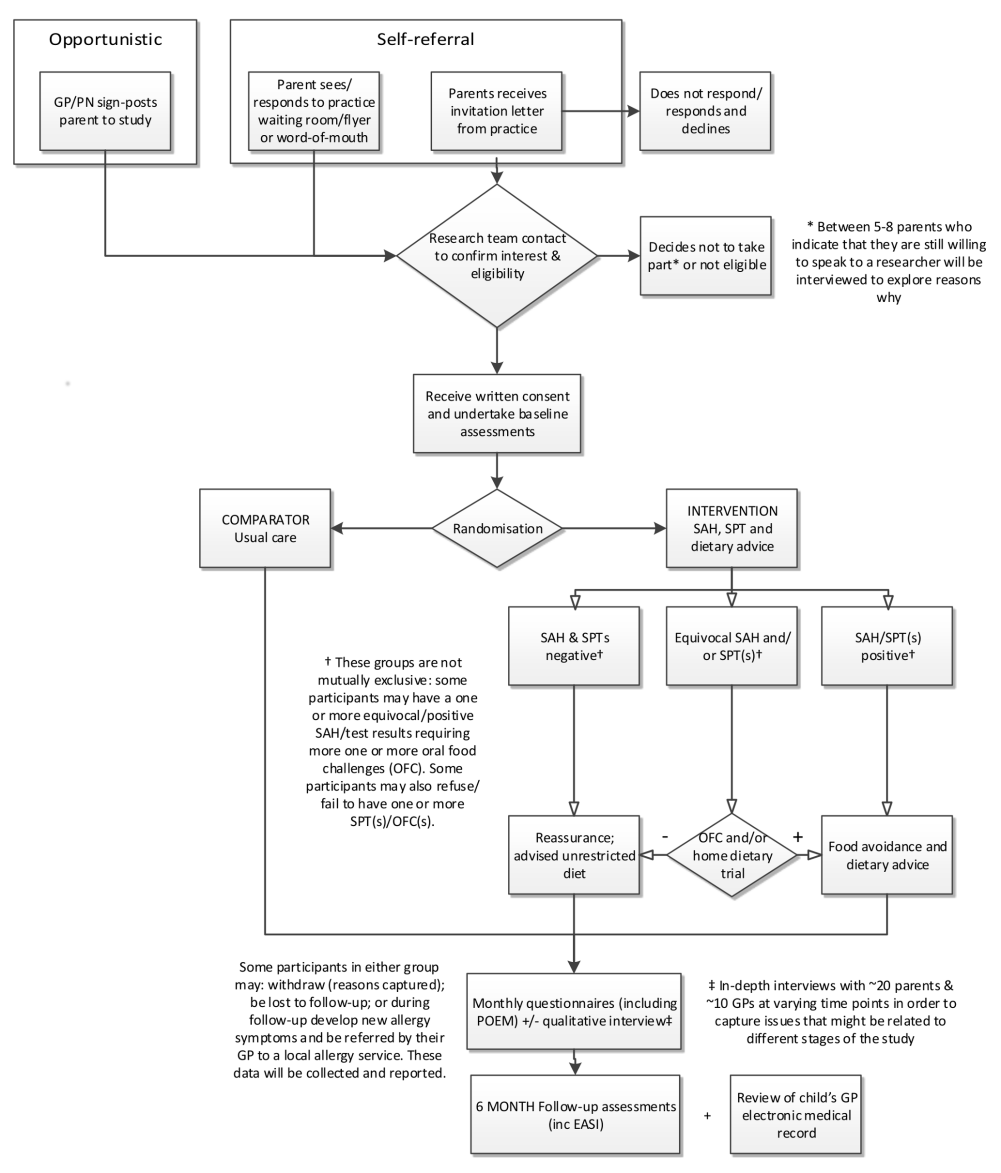
Overview of participant pathway through the study. EASI, Eczema Area Severity Index; GP, general practitioner; PN, practice nurse; POEM, Patient Oriented Eczema Measure; SAH, structured allergy history; SPT, skin prick test.

The stages of participant recruitment are shown in figure 1.

We will identify children aged between 3 months and 5 years with eczema via an electronic query-based records search developed by the research team and run by practice staff at the GP surgeries. A GP or a delegated member of the practice team will screen the search results for inclusion/exclusion criteria and any other known adverse medical or social circumstances that would make invitation to the study inappropriate. Surgeries will be asked to provide the research team with the number of participants excluded, along with a brief reason for exclusion. Parents of potentially eligible children will be sent an invitation pack, comprising an invitation letter, study flyer and response to the invitation to participate form. In addition, we will also recruit participants opportunistically, by placing posters in participating GP surgeries and supplying study flyers for practice staff and health visitors to hand out.

Interested families will be asked to complete a brief screening questionnaire that the research team will use to assess initial eligibility. Parents of potentially eligible participants will be contacted by a member of the research team to explain more about the study and schedule a baseline assessment at a participating GP surgery. At this visit, consent will be received, baseline data collected and randomisation undertaken.

### Eligibility and allocation

Inclusion and exclusion criteria are summarised in box 2.

Box 2 **Participant eligibility criteria**

Inclusion criteria are:

- Child aged between 3 months and 5 years with eczema diagnosed by an appropriately qualified healthcare professional (registered doctor, nurse or health visitor).
- Patient Oriented Eczema Measure Score of >2.
- Consent given by a person with parental responsibility for the participant.

Exclusion criteria are:

- Child with medically diagnosed food allergy, awaiting referral/investigations for possible food allergy or had previous investigations for food allergy (not including home testing).
- The person responsible for consent has insufficient written English to complete the outcome measures, or has another child already taking part in the trial.

Individuals will be randomised to intervention or comparator groups (1:1 ratio), stratified by age (less than 1 year, 1 year to less than 2 years, 2 years and above) and eczema severity (mild, moderate/severe)^14^ and blocked within strata, using the Bristol Randomised Trials Collaboration web-based system. Allocation concealment will be ensured, as the clinical studies officer (CSO) will not randomise the participant until all baseline measurements have been completed.

### Interventions

All participants allocated to the intervention group will undergo a structured allergy history, skin prick tests (SPTs) and will be given dietary advice. Where the child’s history and the results of the SPT results are equivocal, participants will be offered repeat SPTs and/or oral food challenges (OFCs) and/or home dietary trial of exclusion or inclusion. Repeat SPTs will be done either at the same appointment or 12 weeks after the baseline appointment. Advice will be tailored accordingly for mothers who are breast feeding and/or babies who have not yet been weaned.

- **Structured allergy history:** The researcher (CSO) will first take a structured allergy history. There are recommendations for what a structured allergy history should comprise,^15^ but no validated questionnaires. With reference to published guidance,^7 16^ we have therefore modified questionnaires developed for the Barrier Enhancement for Eczema Prevention (BEEP) Trial.^17^ These questions capture relevant symptoms (skin, respiratory and gastrointestinal) and timing of onset in relation to ingestion of the study foods.
- **SPTs:** The CSO will carry out the SPTs using commercial extracts of cow’s milk, hen’s egg (white), wheat, peanut, cashew and codfish, along with positive (1.0% histamine) and negative (0.9% saline) controls.^18 19^One millimetre shouldered sterile lancets will be used (ALK, Denmark) and the diameter (mean of longest and shortest perpendicular axis if ovoid or irregular) of any weal reaction, resulting from the release of histamine and other mediators, will be measured after 15 min.^20^
- **OFC:** Supervised open food challenges will be undertaken at Bristol Royal Children’s Hospital, using a modified Practical Allergy (PRACTALL) dosing schedule and criteria for interpretation of challenge outcome,^21^ usually within 1–2 weeks of the baseline appointment. Consent specifically for OFC will be received and standard hospital protocols for each allergen will be followed. For pragmatic and cost reasons, they will be unblinded as in normal clinical practice, rather than the diagnostic ‘gold standard’ of the double-blind, placebo-controlled food challenge.^22^
- **Home dietary trial:** For participants whose history and investigation findings suggest the possibility of a delayed-type reaction, they will be advised to either exclude or reintroduce (as appropriate to their path in the study) the possible allergen from/into their diet over a period of 2–4 weeks, as per current clinical practice.^16^
- **Dietary advice:** An algorithm describing the approach to the interpretation of the structured allergy history, SPT results, ±OFC, and consequent dietary guidance, will be developed and tested as part of this feasibility study, guided in part by published guidance on diagnosis of food allergy in epidemiological studies.^23^ All participants’ results will also be reviewed by an expert allergy panel and dietary advice relayed to their family accordingly.

Participants in the comparator group will receive care as usual, as described in the National Institute for Health and Care Excellence (NICE) eczema and allergy in children guidelines and will not receive any additional assessments or tests.^16 24^ Any allergy tests and subsequent advice will be monitored as part of this feasibility study.

Regardless of allocation, all care after randomisation, including investigations and/or referrals for possible food allergies, will remain with the participant’s GP.

### Outcomes

The primary outcome is the feasibility of conducting the trial (recruitment, retention, contamination) and collecting the required data (online supplementary appendix 1). A complete schedule of data collection can be found in table 1. The feasibility of collecting data in the key domains that are likely to be used in the definitive trial (symptoms, clinical signs, long-term control and quality of life, as recommended by the core outcome group for eczema, HOME)^25^ will be assessed:

10.1136/bmjopen-2018-028428.supp1 Supplementary file 1

**Table 1:** Schedule of enrolment, interventions and assessments

Week	Study period									Closeout
	Enrolment	Allocation	Postallocation						V_1_	
		V_0_	Follow-up questionnaires							
		0	4	8	12	16	20	24	24	
Parent-completed										
Screening questionnaire	●									
Demographics and medical history		●								
POEM	●	●	●	●	●	●	●	●		
Other eczema symptoms†		●	●	●	●	●	●	●		
Other possible symptoms of food allergy				●				●		
Diet of child (and breastfeeding mother)			●	●	●	●	●	●		
Health service utilisation			●	●	●	●	●	●		
Out-of-pocket expenses/time off work			●	●	●	●	●	●		
ADQoL		●		●				●		
CHU-9D		●		●				●		
IDQoL		●		●				●		
Parental anxiety (GAD-7)		●						●		
Exit questionnaire								●		
Researcher-administered										
UK diagnostic criteria for atopic dermatitis		●								
Other possible symptoms of food allergy		●								
Diet of child (and breastfeeding mother)		●								
EASI		●							●	
Structured allergy history		○								
Skin prick test (SPT)		○								
Oral food challenge (OFC)		*								
Home dietary trial		*								
EMR notes review										●

V_0_=baseline visit; V_1_=follow-up visit at 24 weeks.

●All participants; ○ Only participants in intervention group.

* only participants in intervention group with equivocal structured allergy history/SPT results.

† bother score, itch intensity, parent global assessment.

ADQoL, Atopic Dermatitis Quality of Life; CHU-9D, Children’s Health Utility 9D; EASI, Eczema Area Severity Index; EMR, electronic medical records; GAD-7, Generalised Anxiety Disorder 7; IDQoL, Infant Dermatitis Quality of Life; POEM, Patient Oriented Eczema Measure.

- Patient Oriented Eczema Measure^26^ (POEM, proposed primary outcome in the definitive trial) completed by proxy (parent report) captures symptoms of importance to parents and patients.^27^ Emerging data suggest that monthly, as opposed to weekly, collection is adequate for the purpose of capturing long-term control.^28^ It demonstrates good validity, repeatability and responsiveness to change.^29 30^
- Eczema Area Severity Index (EASI),^31^ a validated scoring system that grades the physical signs of eczema. Administered by a trained researcher, it will provide an independent assessment of eczema severity.
- Long-term control will be captured by repeated, 4 weekly, administration of POEM.
- Disease-specific (Atopic Dermatitis Quality of Life, ADQoL;^32^ Infant Dermatitis Quality of Life^33 34^ and generic (Children’s Health Utility 9D, CHU-9D^35 36^ quality of life measures will be collected at baseline, week 8 and week 24. The CHU-9D is currently validated for children aged 7 years and over,^37^ so additional guidance notes and validation questions are included.

With consent, participants’ electronic medical records (EMRs) will be reviewed at 24 weeks (from 4 weeks before and for the duration of time in the study) for data on NHS consultations, treatments, referrals for eczema/allergies and relevant prescribed medications.

For participants in the intervention group, the following data will also be collected:

- Structured allergy history.
- Results of SPT±OFC±home dietary trial.

### Data collection methods and retention

Baseline data will be collected by the CSO using paper case report forms (CRFs). Parents will be given the option of completing follow-up questionnaires either online or on paper. In recognition of participant’s time and to encourage retention in the study/data collection, parents of participants will be offered £10 vouchers at the baseline and around the 24 weeks visit. We will also offer the child a small gift of about £5 in value.

### Blinding

It is not possible to blind participants, their families or treating clinicians to allocation. The research team will notify the appropriate GP surgery of the participant’s allocation and the outcome of any tests/investigations and food allergy diagnoses.

The CSO undertaking the baseline visit cannot be blinded, but all baseline data (including EASI) will be collected before randomisation. If possible, the follow-up visit will be done by a different CSO, who will be blinded to allocation. Parents will be asked not to disclose allocation to the CSO doing the follow-up visit. CSO blinding will be monitored by means of self-report.

### Participant time line

Participants are in the study for 24 weeks, from the baseline until the follow-up visit. Figure 1 provides an overview of the participants’ pathway through the study.

### Sample size

As this is a feasibility RCT, a formal sample size calculation is not appropriate. On a pragmatic basis, we have determined that 80 children (approximately 40 in each group) will be sufficient to provide estimates of recruitment, retention, adherence and assessment of contamination within GP surgeries and between groups. This is broadly in line with published ‘rules of thumb’.^38 39^

### Data management

Data will be entered onto the study database. The system will incorporate data entry and validation rules to reduce data entry errors, and management functions to facilitate auditing and data quality assurance.

### Statistical methods

The aim will be to determine the feasibility of undertaking the main trial and explore acceptability. We will report our findings following the pilot and feasibility extension of the Consolidated Standards of Reporting Trials (CONSORT) guidance (2010), including a CONSORT diagram, descriptive and summary statistics, along with all important harms or unintended effects in each group.

Descriptive statistics will be used to compare recruitment, retention, adherence and contamination rates overall and between the two groups; and in the intervention group, test results and adherence to dietary advice. Completion rates, average score and distributions (as appropriate) will be reported for the proposed outcomes in the main trial, for example, POEM and EASI.

### Economic scoping

We will gather data on key costs and outcomes to assess the feasibility of carrying out a cost-effectiveness analysis from the primary perspective of the NHS and from a wider perspective including parental costs and time off work.

Data on healthcare contacts and prescribed medications will be extracted from EMRs. Additional healthcare contacts, information about parental out-of-pocket expenses and time off work will be collected using 4-weekly parent-completed questionnaires. The overall level of missing data will be recorded and the pattern of missing data, by item, will be explored. Relevant unit costs will be identified and, once resource-use has been costed, we will identify which items are important cost drivers. The resources required for the intervention will be identified and the feasibility of costing these established.

NICE recommends the use of quality-adjusted life-years (QALYs) as the preferred outcome measure in economic evaluations, but it is unclear what the most appropriate underlying measure is for this population in estimating QALYs. Therefore, we will test feasibility and validity of using both condition-specific (ADQoL)^32^ and generic (CHU-9D)^35 36^ preference-based health-related quality of life measures in children (measured at baseline, 8 weeks and 24 weeks) to estimate QALYs. The CHU-9D is currently validated for children aged 6 years and over, with pilot versions for those aged 5–7 years and additional guidance notes and validation questions for those under 5 years. One key component of the economic work will be to determine the feasibility of using the CHU-9D in this preschool age group.

### Nested qualitative study

The aims of the qualitative study are to help interpret and explain the quantitative feasibility findings (including experience and acceptability of study processes/interventions); and to generate new knowledge around the issues of food allergy, allergy tests and dietary modification in children with eczema, from the perspective of parents and GPs.

GPs at participating surgeries will be asked to complete a brief questionnaire and all parents and GPs will be asked whether they are willing to be contacted to take part in an interview. Semistructured qualitative interviews will be conducted with a sample of trial parents and GPs from participating surgeries, using topic guides developed based on study aims and input from the Trial Management Group (TMG).

Parents will be selected purposively to ensure diversity in relevant characteristics: trial group (intervention or comparator) with oversampling of the intervention group; eczema severity according to POEM (mild/moderate [<17] vs severe [≥17]); socioeconomic status (assessed via postcode, using the Index of Multiple Deprivation Decile [categories: high [8–10]/medium [5–7]/low [1–4]]);^40^ for mothers, whether currently breast feeding; and length of time in the trial (shortly after baseline visit or OFC, or later in the trial). GPs will be purposively sampled to capture variation in GP surgery deprivation decile,^40^ length of time in the trial, number of years’ experience as a GP and confidence in managing children with eczema (assessed via a single item scored: 1 [low] to 10 [high]). Sampling will stop when we have sufficient information power relevant to the study aims;^41^ we anticipate a total of 20 parent and 10 GP interviews.

In addition, we will conduct brief telephone interviews with 5–8 parents who are ineligible to participate, decline to take part, or withdraw during the trial but indicate that they are willing to discuss reasons why. This information may provide valuable data to inform the design of a future definitive trial.

Interviews will be conducted by an experienced qualitative researcher, either by telephone or face to face, depending on the preference of the interviewee, audio-recorded (with permission) and transcribed verbatim. All interviewees will receive an information sheet and consent form to read in advance of the interview. Written informed consent will be taken prior to face-to-face interviews, and verbal consent will be taken for telephone interviews.

Data analysis of interview transcripts will take place alongside data collection and inform further data collection. We will conduct a thematic analysis, using both inductive and deductive coding (informed by the Common Sense model).^42^

### Monitoring, safety and audit

Because this is a low-risk feasibility trial, the trial is overseen by a joint Trial Steering/Data Monitoring Committee (TS/DM-C) which comprises four independent members: a chairperson, a biostatistician, a clinician and a patient representative (parent of child with eczema). Their role will be to provide overall supervision of the trial on behalf of the funder, with a focus on progress of the trial, adherence to the protocol, patient safety and consideration of new information.

Adverse events will be collected in the CRFs and by parent/clinician report and reported to the TMG and TS/DM-C. Possible serious adverse events include:

- Severe localised reaction (redness, swelling, itch) to one or more SPTs necessitating medication and/or hospitalisation.
- Anaphylactic reaction (generalised flushing of the skin, hives, swelling of throat and mouth, difficulty in swallowing or speaking, tachycardia, severe asthma, abdominal pain and/or nausea and vomiting, hypotension and/or collapse and unconsciousness) requiring medication±hospitalisation (SPTs or OFC).

The sponsor organisation is the University of Bristol.

## Pregrant application survey

An online survey of parents of children with eczema informed the study design. It was promoted via social media and partner eczema and allergy websites between 10 October 2016 and 27 October 2016. We received 152 responses, 97% (145/150) female with a mean age of 38.8 years. The median number of children with eczema was 1 (IQR 1, 2) and the mean POEM score (for the worst-affected child, with more than one child with eczema) was 11.7 (SD 7.6). Seventy-four per cent (108/146) had one or more food allergies, the most common being peanut, egg and cow’s milk; 71.3% (77/108) had received allergy tests and had been given advice by a healthcare professional and 17.6% (19/108) based their report on their observation of symptoms/reaction alone.

Participants were asked ’In a study that compares the effect of doing allergy tests or giving advice on avoiding certain foods in children, what would be the single most important thing that this kind of study could tell you about?’ Overall, 37% (56/151) chose ‘Reduce the risk of a sudden or severe allergic reaction’. However, among those children without a reported food allergy (the group of interest in this study), 44% (16/36) chose ‘Reduce day-to-day severity of eczema’. Consequently, we included eczema severity as a key clinical outcome.

Regarding the then proposed study, 96% (144/150) said they would be willing for their child to have an allergy test, with 67.1% (100/149) identifying skin prick as their ‘first choice’ option for testing for allergy and 54.3% (82/151) saying a blood test was an acceptable ‘second choice’. Other participants said they would refuse (4.0% skin prick, 8.5% blood test) or did not know (2.0% skin prick, 2.6% blood test). Further information about the limitations of both types of tests (risk of false reassurance or worry) did not change the opinion of the majority (72.5%, 108/149) of respondents; 56.9% (74/130) said that based on the clinical history and allergy test, they would be willing to avoid that food for at least 24 months. These findings provided reassurance as to the acceptability of the intervention, which includes SPTs and the possibility of having to exclude foods for at least several months.

## Public and patient involvement

The James Lind Alliance Eczema Priority Setting Partnership (2013) identified the following questions: ‘What role might food allergy tests play in treating eczema?’ and ‘What is the role of (exclusion) diets in treating eczema?',^43^ which a follow-on definitive trial could begin to address.

Two mothers of children with eczema (Catherine Gray and Jo McMeechan) are members of the TMG and regularly attend the meetings. They have commented on the research proposal and study paperwork, and their suggestions around nomenclature and reducing data burden on participants have been incorporated. A lay member also sits on TS/DM-C.

We have established and met with a wider PPI advisory group. It first met towards the beginning of the research to discuss data burden and the design of patient-facing materials. At a subsequent meeting, study progress and challenges were discussed. One more meeting is planned towards the end of the study, to inform write-up and dissemination of findings.

## Ethics and dissemination

### Protocol amendments

Any amendments to the protocol will be reported accordingly to the regulatory bodies, with a full copy of the current protocol available for download from the study website. Amendments to date are listed in online supplementary appendix 1.

### Consent or assent

Written consent for taking part in the trial will be received by a CSO from the parent or guardian of the participant at their baseline appointment, which takes place in a participating GP practice. Consent is also sought to contact participants regarding possible interview in the nested qualitative study; and for the reuse of the anonymised data in future research for purposes not related to this study, including as publicly available ‘open data’ (see online supplementary appendix 2). Consent for OFCs is received by the hospital nurse undertaking the procedure.

### Confidentiality and access to data

The database and randomisation system will protect patient information in line with the data protection legislation. Trial staff will ensure that participants’ anonymity is maintained through protective and secure handling and storage of patient information at the trial centre.

The chief investigator will have access to and act as custodian of the full data set, which will be made available to the TS/DM-C if requested to verify the validity of the findings.

### Ancillary and post-trial care

Participants requiring follow-up beyond their 6 months in the study will be referred by their GP to their local allergy clinic.

### Data sharing

Study progress, outputs and a summary of findings will be made available via a study website and Twitter account; and summaries distributed to participating families and GP surgeries.

No later than 3 years after the completion of the study, we will make available a completely deidentified data set to an appropriate data archive for sharing purposes.

## Discussion

There are wide variations in provision of allergy testing for children with eczema. Parental concern and clinician uncertainty about the role of food allergy in eczema has been highlighted as a barrier to effective treatment.^44^ Up to 70% of parents make significant modifications to their child’s diet, often without professional advice,^45^ even if the child has only mild eczema. Many parents turn to the internet for advice,^12 46^ or purchase self-test allergy kits which are not validated and not recommended.^16^

It is uncommon for allergy tests to be undertaken in primary care but in principle, allergy testing (in the form of SPTs) and advice could be routinely delivered in primary care, but evidence is required to demonstrate both the feasibility and value of doing so. An RCT is needed to determine the clinical effectiveness and cost effectiveness of food allergy testing and advice in primary care, on severity of eczema in children. There are potentially significant benefits for the NHS of improving long-term eczema management, avoiding serious allergic reactions, and targeting child nutrition. This study will provide important data to first, determine the feasibility of a large, definitive trial; and second, to inform its design.

The full/most up-to-date version of the protocol is available to download from the study website. The first participant was randomised in September 2018 and recruitment is ongoing. Follow-up is expected to be complete by September 2019. We expect to report in early 2020.

10.1136/bmjopen-2018-028428.supp2 Supplementary file 2

## Supplementary Material

Reviewer comments

Author's manuscript
