# Supplementary material for: TEST (Trial of Eczema allergy Screening Tests): protocol for feasibility randomised controlled trial of allergy tests in children with eczema, including economic scoping and nested qualitative study
Source: BMJ Open. 2019 May 9;9(5):e028428. doi: 10.1136/bmjopen-2018-028428 (PMC6527983; doi:10.1136/bmjopen-2018-028428)
Supplement: Supplementary file 1 [file bmjopen-2018-028428supp001.pdf]

Appendix 1: Administrative information

Title

TEST (Trial of Eczema allergy Screening Tests): a single centre, individually randomised, two-group feasibility randomised controlled trial of allergy tests in children with eczema, with economic scoping and nested qualitative study

Trial registration number

ISRCTN: 15397185 (30 July 2018)

World Health Organization Trial Registration Data Set

Data category	Information
Primary registry and trial identifying number	ISRCTN 15397185
Date of registration in primary registry	30 July 2018
Secondary identifying numbers	IRAS: 237046<br>NHS REC: 18/WM/0124
Source(s) of monetary or material support	NIHR School for Primary Care Research
Primary sponsor	University of Bristol
Secondary sponsor(s)	Not applicable
Contact for public queries	Mr Doug Webb, test-study@bristol.ac.uk, 0117 928 7351
Contact for scientific queries	Dr Matthew Ridd FRCGP PhD, m.ridd@bristol.ac.uk, 0117 331 4557
Public title	Trial of Eczema allergy Screening Tests (TEST)
Scientific title	The TEST (Trial of Eczema Allergy Screening Tests) study: feasibility randomised controlled trial with economic scoping and nested qualitative study
Countries of recruitment	England
Health condition(s) or problem(s) studied	Childhood eczema
Intervention(s)	Structured allergy history and skin prick tests; depending on outcome of these tests, reassurance, repeat skin prick test(s), oral food challenge and/or home dietary trial of exclusion or inclusion
Key inclusion and exclusion criteria	Inclusion: aged between 3 months and less than 5 years; eczema diagnosed by an appropriately qualified healthcare professional; mild, moderate or severe eczema (Patient Orientated Eczema Measure (POEM) score>2)<br>Exclusion: medically-diagnosed food allergy or awaiting referral/investigations for possible food allergy; previous investigations for food allergy (does not include home testing)
Study type	Intervention

Date of first enrolment	12 September 2018
Target sample size	80
Recruitment status	Recruiting
Primary outcome(s)	The feasibility of conducting the trial (recruitment, retention, contamination) and collecting the required data: recruitment and retention rates; acceptability of recruitment, intervention and follow-up procedures to parents/carers; acceptability of trial processes and procedures to GPs; development and refinement of a manual on the interpretation of test results and dietary advice to be given; number of participants In the intervention group with positive/negative tests; adherence to dietary advice; contamination of the control group; acceptability and feasibility of collecting clinical outcomes; feasibility and optimise collection of patient-level data on NHS and personal resource use; feasibility of using the CHU-9D in children under 5 years of age; inform eligibility criteria for the future definitive trial; detection bias in the collection of patient-reported outcomes; trial processes and logistics
Key secondary outcomes	Eczema symptoms, measured using POEM; eczema signs, measured using EASI; eczema 'bother' score; itch intensity score; parent global assessment of eczema; other possible symptoms of food allergy; UK diagnostic criteria for atopic dermatitis; main carer anxiety, measured using GAD-7; diet of child and/or mother if child being breastfed by her; adverse events; child and family quality of life, measured using ADQoL, CHU-9D and IDQoL; satisfaction with trial processes, procedures and paperwork; health services utilisation; out-of-pocket expenses/time off work.

Protocol version

Version 2.0 (18 October 2018)

Version		Notes
**Number**	**Date**	
2.0	18.10.18	Section 5.2: addition of missing data collection points (Diet of child and breast-feeding mother at baseline; ADQoL at 8 weeks)<br><br>Section 8.4: change "avoidance of food(s) with dietary advice; and referral via GP for follow-up" to "avoidance of food(s) with dietary advice; and referral to the local NHS allergy services via GP for longer-term follow-up" ; and "Any participants with indeterminate results will be reviewed by an expert allergy panel (co-applicants Boyle & Marriage) …" to "All participants' results will be reviewed by an expert allergy panel (including co-applicants Ridd, Boyle, Marriage and/or Waddell) …"<br><br>Section 9.1: change "Up to 12 GP surgeries" to "At least 12 GP surgeries …";<br><br>Section 9.3 & section 10.2: change "in/at their [own] GP practice" to "at a participating GP practice";<br><br>Section 10.1: description of expression of interest form corrected from "The form will comprise which will comprise POEM, questions asking their opinion of the role of diet/food allergy in their child's eczema, and any

		previous food allergy tests, diagnoses and/or dietary modifications" to "The form asks if they currently have eczema/a medically diagnosed food allergy and the POEM questions"<br><br>Section 11.3: Change from "In addition, we will conduct brief telephone interviews with ~5-8 parents who decline to take part in response to the initial invitation letter or later withdrawal from the trial but indicate that they are willing to discuss reasons why" to "In addition, we will conduct brief telephone interviews with ~5-8 parents who are ineligible, decline to take part or withdrawal from the trial but indicate that they are willing to discuss reasons why"<br><br>Section 16.1: change "Expected SAEs defined in the study protocol (page 39) …" to "Expected SAEs as defined below …"<br><br>Section 18.1: revised project duration/milestones<br><br>Other minor changes (correction of typing errors, changes in research team)
1.0	29.03.18	Submitted/approved by REC/HRA

Funding

NIHR School for Primary Care Research

Contributorship

See main manuscript

Sponsor contact information

Trial sponsor: University of Bristol
Sponsor's reference: 2832
Contact name: Mrs Anna Brooke
Address: Research Enterprise Development, One Cathedral Square, Bristol BS1 5DD
Email: research-governance@bristol.ac.uk
Telephone: 0117 428 4011

Role of study sponsor and funder

The funder and sponsor had no role in the design of this study and will not have any role during its execution, analyses, interpretation of the data, or decision to submit results.

Committees

The Trial Management Group (TMG) comprises all investigators, the trial manager, research and administrative staff, with input from patient/public representatives. Members of the TMG will contribute to the trial in the following ways: trial design and methods; participant recruitment and trial conduct; trial management; trial logistics and cost management; economic evaluation; qualitative study statistical data analysis; and publication. The TMG will meet on a regular basis to oversee the management of the trial. The TMG will be provided with detailed information by the centre staff regarding trial progress. Meetings will be face-to-face with teleconference facilities for TMG members who are unable to be present.

This study was designed and is being delivered in collaboration with the Bristol Randomised Trials Collaboration (BRTC), a UKCRC registered clinical trials unit which, as part of the Bristol Trials Centre, is in receipt of National Institute for Health Research CTU support funding. Members of the BRTC will attend the TMG.

Because this is a low-risk trial, the funder has agreed that the roles of both guiding the Trial Management Group and monitoring trial data will be undertaken by a single Trial Steering/Data Monitoring Committee (TS/DM-C). The TS/DM-C will meet at least three times over the course of the study and comprises four independent members: a chairperson, a biostatistician, a clinician, and a patient representative (parent of child with eczema). Their role will be to provide overall supervision of the trial on behalf of the funder, with a focus on progress of the trial, adherence to the protocol, patient safety and consideration of new information. The committee will review the accruing data and assess whether there are any safety issues that should be brought to the Sponsor's or the participants' attention or any reasons for the trial not to continue. Terms of reference will be drawn up and agreed with members of the TS/DM-C.
