# Supplementary material for: TEST (Trial of Eczema allergy Screening Tests): protocol for feasibility randomised controlled trial of allergy tests in children with eczema, including economic scoping and nested qualitative study
Source: BMJ Open. 2019 May 9;9(5):e028428. doi: 10.1136/bmjopen-2018-028428 (PMC6527983; doi:10.1136/bmjopen-2018-028428)
Supplement: Supplementary file 2 [file bmjopen-2018-028428supp002.pdf]

University of Bristol | University of Southampton | The University of Nottingham (United Kingdom · China · Malaysia) | Bristol Randomised Trials Collaboration

The Trial of Eczema allergy Screening Tests (TEST) Study

Parent/Carer Consent Form

Initial box

1. I confirm that I have read and understand the Participant Information Sheet dated 18.10.18 Version 2.0 for the above study. I have had the opportunity to consider the information, ask questions and have had these questions answered satisfactorily. ☐
2. I understand that participation is voluntary and that we are free to withdraw at any time without giving any reason, without my child's medical care or legal rights being affected. ☐
3. I understand that after the study ends, the data collected will be made "open data". I understand that this means the anonymised data will be publicly available and may be used for purposes not related to this study. I understand that it will not be possible to identify me from these data. ☐
4. I understand that relevant sections of my child's medical notes and all information collected for this research may be reviewed by the study team, by the participating NHS Trust to ensure that the research is conducted appropriately. I give permission for these individuals to access my child's records as appropriate. ☐
5. I agree that my child's family doctor (GP) will be told that they are taking part in the study ☐
6. I give consent for the data collected in this trial to be used in future ethically approved studies on the understanding that all information will continue to be kept securely and remain confidential. ☐
7. I give consent to be contacted by a member of the research team with a view to being interviewed about my opinions about allergy testing for eczema and taking part in TEST. I understand that if I am contacted, I will be given more information about the interviews by the research team, I can decide later about taking part and I understand I will be asked to give further consent. ☐
8. I agree for myself and my child to take part in the above-named study. ☐

Name of Participant (Child)	Participant ID	
Name of Parent/Guardian	Signature	Date
Name of person receiving consent	Signature	Date

When completed: 1 (original) for research team, 1 for participant

Funded by NHS National Institute for Health Research

The research was funded by the NIHR Health Technology Assessment (HTA) Programme. The views expressed are those of the author(s) and not necessarily those of the NHS, the NIHR or the Department of Health.
