# Supplementary material for: TEST (Trial of Eczema allergy Screening Tests): protocol for feasibility randomised controlled trial of allergy tests in children with eczema, including economic scoping and nested qualitative study
Source: BMJ Open. 2019 May 9;9(5):e028428. doi: 10.1136/bmjopen-2018-028428 (PMC6527983; doi:10.1136/bmjopen-2018-028428)
Supplement: Author's manuscript [file bmjopen-2018-028428.draft_revisions.pdf]

BMJ Open

BMJ Open is committed to open peer review. As part of this commitment we make the peer review history of every article we publish publicly available.

When an article is published we post the peer reviewers' comments and the authors' responses online. We also post the versions of the paper that were used during peer review. These are the versions that the peer review comments apply to.

The versions of the paper that follow are the versions that were submitted during the peer review process. They are not the versions of record or the final published versions. They should not be cited or distributed as the published version of this manuscript.

BMJ Open is an open access journal and the full, final, typeset and author-corrected version of record of the manuscript is available on our site with no access controls, subscription charges or pay-per-view fees (http://bmjopen.bmj.com).

If you have any questions on BMJ Open's open peer review process please email info.bmjopen@bmj.com

BMJ Open

TEST (Trial of Eczema allergy Screening Tests): a single centre, individually randomised, two-group feasibility randomised controlled trial of allergy tests in children with eczema, including economic scoping and nested qualitative study

Journal:	*BMJ Open*
Manuscript ID	bmjopen-2018-028428
Article Type:	Protocol
Date Submitted by the Author:	07-Dec-2018
Complete List of Authors:	Ridd, Matthew; University of Bristol, Social & Community Medicine; University of Bristol<br>Edwards, Louisa; Simon Fraser University, Faculty of Health Sciences; Centre for Clinical Epidemiology and Evaluation (C2E2),<br>Santer, Miriam; University of Southampton, Primary Care & Population Sciences<br>Chalmers, Joanne; University of Nottingham, Dermatology<br>Waddell, Lisa; Nottingham CityCare<br>Marriage, Deborah; Bristol Royal Hospital for Children<br>Muller, Ingrid; University of Southampton, Department of Psychology<br>Roberts, Kirsty; University of Bristol School of Social and Community Medicine<br>Garfield, Kirsty; University of Bristol, Bristol Randomised Trials Collaboration<br>Coast, Joanna; Bristol University, Population Health Sciences<br>Selman, Lucy; University of Bristol School of Social and Community Medicine<br>Clement, Clare; University of Bristol, School of Social and Community Medicine; University of Bristol<br>Shaw, Ali; University of Bristol, School of Social and Community Medicine<br>Angier, Elizabeth; University of Southampton<br>Blair, Peter; University of Bristol, Centre for Academic Child Health<br>Turner, Nicholas; University of Bristol,<br>Taylor, Jodi; University of Bristol, School of Clinical Sciences<br>Kai, Joe; University of Nottingham, Division of Primary Care<br>Boyle, R; Imperial College London
Keywords:	Eczema < DERMATOLOGY, Allergy < THORACIC MEDICINE, Clinical trials < THERAPEUTICS

SCHOLARONE™
Manuscripts

Title page

Title

TEST (Trial of Eczema allergy Screening Tests): a single centre, individually randomised, two-group feasibility randomised controlled trial of allergy tests in children with eczema, including economic scoping and nested qualitative study

Authors

Matthew J Ridd,[1] Louisa Edwards,[2] Miriam Santer,[3] Joanne R Chalmers,[4] Lisa Waddell,[5] Deb Marriage,[6] Ingrid Muller,[2] Kirsty Roberts,[1] Kirsty Garfield,[7] Joanna Coast,[8] Lucy Selman,[7] Clare Clement,[7] Alison Heawood,[1] Elizabeth Angier,[3] Peter S Blair,[9] Nicholas L Turner,[7] Jodi Taylor,[7] Joe Kai,[10] Robert J Boyle[11]

[1] Population Health Sciences, University of Bristol

[2] Faculty of Health Sciences, Simon Fraser University, Burnaby, Canada

[3] Primary Care and Population Sciences, University of Southampton, Southampton, UK

[4] Centre of Evidence Based Dermatology, University of Nottingham, Nottingham, UK

[5] Nottingham CityCare Partnership, Nottingham, UK

[6] Bristol Royal Hospital for Children, University Hospitals Bristol NHS Foundation Trust, Bristol, UK

[7] Bristol Randomised Trials Collaboration, Bristol Trials Centre, University of Bristol, Bristol, UK.

[8] Health Economics at Bristol, Population Health Sciences, University of Bristol, UK

[9] Centre for Academic Child Health, Population Health Sciences, University of Bristol, UK

[10] Division of Primary Care, University of Nottingham, UK

[11] Section of Paediatrics, Imperial College London, London UK; Centre of Evidence Based Dermatology, University of Nottingham, Nottingham, UK

Corresponding author

Matthew Ridd, GP and Reader in Primary Healthcare

Postal address: Centre for Academic Primary Care, Population Health Sciences, Bristol Medical School, University of Bristol, Canynge Hall, 39 Whatley Road, Bristol BS8 2PS

Email: m.ridd@bristol.ac.uk

Telephone: 0117 331 4557

Word count

excluding title page, abstract, references, figures and tables: 3984/4000

Keywords

Atopic eczema/dermatitis, food allergy, feasibility RCT

For peer review only - http://bmjopen.bmj.com/site/about/guidelines.xhtml

For peer review only

ABSTRACT

Background

Early-onset eczema is associated with food allergy, and allergic reactions to foods can cause acute exacerbations of eczema. Parents often pursue dietary restrictions as a way of managing eczema and seek allergy testing for their children to guide dietary management. However, it is unclear whether test-guided dietary management improves eczema symptoms, and whether the practice causes harm through reduced use of conventional eczema treatment or unnecessary dietary restrictions. The aim of the TEST (Trial of Eczema allergy Screening Tests) study is to determine the feasibility of conducting a trial comparing food allergy testing and dietary advice versus usual care, for the management of eczema in children.

Methods and analysis

Design: a single centre, two-group, individually randomised, feasibility RCT with economic scoping and a nested qualitative study. Setting: GP surgeries in the West of England. Participants: children aged over 3 months and less than 5 years with mild to severe eczema. Interventions: allergy testing (structured allergy history and skin prick tests) or usual care. Sample size and outcome measures: we aim to recruit 80 participants and follow them up using 4-weekly questionnaires for 24 weeks. Nested qualitative study: We will conduct ~20 interviews with parents of participating children, 5-8 interviews with parents who decline or withdraw from the trial and ~10 interviews with participating GPs. Economic scoping: We will gather data on key costs and outcomes to assess the feasibility of carrying out a cost-effectiveness analysis in a future definitive trial.

Ethics and dissemination

The study has been reviewed by the Health Research Authority and given a favourable opinion by the NHS REC (West Midlands – South Birmingham Research Ethics Committee, Reference Number 18/WM/0124). Findings will be submitted for presentation at conferences and written up for publication in peer-reviewed journals.

Trial registration

ISRCTN: 15397185 (30 July 2018)

Word count: 296/300

Strength and limitations

- This is the first RCT exploring test-guided dietary management for treating eczema to be done in a primary care setting, where most children with eczema are diagnosed and managed in the UK.
- Data on the processes and outcomes that are being collected will help determine the feasibility of a definitive trial and associated economic evaluation.
- The nested qualitative study will help to interpret and explain the quantitative feasibility findings and to generate new knowledge around the issues of food allergy, allergy tests and dietary modification in children with eczema, from the perspective of parents and GPs
- The study is being conducted in a single centre in the West of England, which may limit the generalisability of the findings.

For peer review only - http://bmjopen.bmj.com/site/about/guidelines.xhtml

Main text

INTRODUCTION

Background and rationale

Childhood eczema is a common long-term condition characterised by dry and itchy skin. In accordance with the recommended nomenclature of the World Allergy Organisation, we use the label "eczema" to refer to the clinical phenotype of atopic eczema/dermatitis.[1]

Eczema affects around 20% of pre-school age children; 60% of these develop symptoms in the first year of life and 90% by five years of age.[2] In the UK. most children with eczema are diagnosed and managed in primary care with a combination of emollients and topical corticosteroids. Having eczema can significantly impact the quality of life of the affected child and their family. Treatment adherence can be problematic for numerous reasons, including parents/carers (hereafter, "parents") seeking a "cure" through dietary exclusions for possible food allergy rather than "control" through long-term use of topical treatments.[3-5]

Eczema is associated with food allergy, especially early-onset, troublesome eczema,[6] and parents of children with eczema often try dietary exclusions in an attempt to reduce symptom severity and may seek allergy testing to guide such dietary exclusions. Allergic reactions to food can cause an acute exacerbation of eczema, either as part of an IgE-mediated reaction or as an isolated non-IgE mediated reaction to a food (see Table 1). Parents' suspicions of food allergies in general and especially with respect to eczema have low specificity. Depending on the specific population studied and the definitions used, 15-36% of children with eczema compared to about 6% of the general population have a food sensitivity (a 'positive' test result, without clinical symptoms) or allergy.[7] Clinical practice in offering allergy tests to parents of children with eczema varies significantly, with many allergy clinics routinely "screening" for associated food allergies, but few primary care services offering testing in the absence of a history suggesting an IgE-mediated reaction to a food.

A Cochrane review[8] of dietary exclusions for adults and children with eczema published in 2008 did not find any evidence of benefit for exclusion diets in unselected populations (i.e. those without clinically suspected food allergies), but did identify one trial which suggested that infants with suspected egg allergy who have positive specific IgE to eggs may benefit from an egg-free diet.[9] While this suggests that test-guided dietary management may be worthwhile, both this and two other subsequently published systematic reviews[10 11] have called for better-designed and conducted trials. We have not identified any economic evaluations in this area and while concerns about food allergy have been raised during in-depth interviews of parents' general experiences of looking after children with eczema,[3-5] and have arisen as an important concern for parents in online discussion forums,[12] we are not aware of any qualitative work specifically exploring this issue.

Aim and objectives

The aim of the study to determine the feasibility of conducting a trial comparing test-guided dietary management versus usual care, for the management of eczema in children.

The objectives are to explore the following factors that will determine the feasibility and inform the design of a future, full-scale clinical and cost-effectiveness RCT:

- participant recruitment (including numbers potentially eligible), retention and adherence to allocation/dietary advice;
- outcome completion rates; and

For peer review only - http://bmjopen.bmj.com/site/about/guidelines.xhtml

- logistics of of trial processes and their acceptability to participants

Trial design

TEST is a single centre, two-group, individually randomised, feasibility RCT[13] with economic scoping and nested qualitative study.

METHODS AND ANALYSIS

Study setting

Primary care (GP surgeries) in the West of England.

Recruitment

The stages of participant recruitment are shown in **Error! Reference source not found.**.

We will identify children aged between 3 months and 5 years with eczema via an electronic query-based records search developed by the research team and run by practice staff at the GP surgeries. A GP or a delegated member of the practice team will screen the search results for inclusion/exclusion criteria and any other known adverse medical or social circumstance that would make invitation to the study inappropriate. Surgeries will be asked to provide the research team with the number of participants excluded, along with a brief reason for exclusion. Parents of potentially eligible children will be sent an invitation pack, comprising an invitation letter, study flyer and response to invitation to participate form. In addition, we will also recruit participants opportunistically, by placing posters in participating GP surgeries and supplying study flyers for practice staff and health visitors to hand out.

Interested families will be asked to complete a brief screening questionnaire that the research team will use to assess initial eligibility. Parents of potentially eligible participants will be contacted by a member of the research team to explain more about the study and schedule a baseline assessment at a participating GP surgery. At this visit, consent will be received, baseline data collected, and randomisation undertaken.

Eligibility and allocation

Inclusion and exclusion criteria are summarised in Table 2.

Individual randomisation to intervention or comparator groups (1:1 ratio), stratified by age (less than 1 year, 1 year to less than 2 years, 2 years and above) and eczema severity (mild, moderate/severe)[14] and blocked within strata, using the Bristol Randomised Trials Collaboration (BRTC) web-based system. Allocation concealment will be ensured, as the CSO will not randomise the participant until all baseline measurements have been completed.

Interventions

All participants allocated to the intervention group will undergo a structured allergy history, skin prick tests and will be given dietary advice. Where the child's history and the results of the skin prick test results are equivocal, participants will be offered repeat skin prick tests and/or oral food challenges and/or home dietary trial of exclusion or inclusion. Repeat skin prick tests will be done either at the same appointment or 12 weeks after the baseline appointment. Advice will be tailored accordingly for mothers who are breastfeeding and/or babies who have not yet been weaned.

- **Structured allergy history:** The researcher (Clinical Studies Officer, CSO) will first take a structured allergy history. There are recommendations for what a structured allergy history should comprise,[15] but no validated questionnaires. With reference to published guidance,[7,16] we have therefore modified questionnaires developed for the BEEP trial.[17] These

For peer review only - http://bmjopen.bmj.com/site/about/guidelines.xhtml

questions capture relevant symptoms (skin, respiratory and gastrointestinal) and timing of onset in relation to ingestion of the study foods.

- **Skin prick tests:** The CSO will carry out the skin prick tests using commercial extracts of cow's milk, hen's egg (white), wheat, peanut, cashew and codfish, along with positive (1.0% histamine) and negative (0.9% saline) controls.[18 19] 1 mm shouldered sterile lancets will be used (ALK, Denmark) and the diameter (mean of longest and shortest perpendicular axis if ovoid or irregular) of any wheal reaction, resulting from the release of histamine and other mediators, will be measured after 15 minutes.[20]
- **Oral food challenge:** Supervised open food challenges will be undertaken at Bristol Royal Children's Hospital, using a modified PRACTALL dosing schedule and criteria for interpretation of challenge outcome,[21] usually within 1-2 weeks of the baseline appointment. Consent specifically for oral food challenge will be received and standard hospital protocols for each allergen will be followed. For pragmatic and cost reasons, they will be unblinded as in normal clinical practice, rather than the diagnostic "gold standard" of the double-blind, placebo-controlled food challenge.[22]
- **Home dietary trial:** For participants whose history and investigation findings suggest the possibility of a delayed-type reaction, they will be advised to either exclude or reintroduce (as appropriate to their path in the study) the possible allergen from/into their diet over a 2-4 week period, as per current clinical practice.[16]
- **Dietary advice:** An algorithm describing the approach to the interpretation of the structured allergy history, skin prick test results, +/- oral food challenge, and consequent dietary guidance, will be developed and tested as part of this feasibility study, guided in part by published guidance on diagnosis of food allergy in epidemiological studies.[23] All participants' results will also be reviewed by an expert allergy panel and dietary advice relayed to their family accordingly.

Participants in the comparator group will receive care as usual, as described in the NICE eczema and allergy in children guidelines and will not receive any additional assessments or tests.[16 24] Any allergy tests and subsequent advice will be monitored as part of this feasibility study.

Regardless of allocation, all care after randomisation, including investigations and/or referrals for possible food allergies, will remain with the participant's GP.

Outcomes

A complete schedule of data collection can be found in

For peer review only

For peer review only - http://bmjopen.bmj.com/site/about/guidelines.xhtml

Table 3. The feasibility of collecting data in the key domains that are likely to be used in the definitive trial (symptoms, clinical signs, long-term control and quality of life, as recommended by the core outcome group for eczema, HOME)[25] will be assessed:

- Patient Orientated Eczema Measure[26] (POEM, proposed primary outcome in the definitive trial) completed by proxy (parent report) captures symptoms of importance to parents and patients.[27] Emerging data suggests that monthly, as opposed to weekly, collection is adequate for the purpose of capturing long-term control.[28] It demonstrates good validity, repeatability and responsiveness to change.[29 30]
- Eczema Area Severity Index (EASI),[31] a validated scoring system that grades the physical signs of eczema. Administered by a trained researcher, it will provide an independent assessment of eczema severity.
- Long-term control will be captured by repeated, four weekly, administration of POEM.
- Disease-specific (Atopic Dermatitis Quality of Life, ADQoL;[32] Infant Dermatitis Quality of Life, IDQoL[33 34]) and generic (Children's Health Utility 9D, CHU-9D[35 36]) quality of life measures will be collected at baseline, weeks 8 and 24. The CHU-9D is currently validated for children aged 7 years and over,[37] so additional guidance notes and validation questions are included.

With consent, participants' electronic medical records (EMR) will be reviewed at 24 weeks (from four weeks before and for the duration of time in the study) for data on NHS consultations, treatments, referrals for eczema/allergies and relevant prescribed medications.

For participants in the intervention group, the following data will also be collected:

- Structured allergy history
- Results of Skin Prick Test (SPT) +/- Oral Food Challenge (OFC) +/- home dietary trial

Data collection methods and retention

Baseline data will be collected by the CSO using paper case report forms (CRFs). Parents will be given the option of completing follow-up questionnaires either online or on paper. In recognition of participant's time and to encourage retention in the study/data collection, parents of participants will be offered £10 vouchers at the baseline and around the 24-week visit. We will also offer the child a small gift of about £5 in value.

Blinding

It is not possible to blind participants, their families or treating clinicians to allocation. The research team will notify the appropriate GP surgery of the participant's allocation and the outcome of any tests/investigations and food allergy diagnoses.

The CSO undertaking the baseline visit cannot be blinded, but all baseline data (including EASI) will be collected before randomisation. If possible, the follow-up visit will be done by a different CSO, who will be blinded to allocation. Parents will be asked not to disclose allocation to the CSO doing the follow-up visit. CSO blinding will be monitored by means of self-report.

Participant timeline

Participants are in the study for 24 weeks, from the baseline until the follow-up visit. **Error! Reference source not found.** provides an overview of participants' pathway through the study.

Sample size

As this is a feasibility RCT, a formal sample size calculation is not appropriate. On a pragmatic basis, we have determined that 80 children (approximately 40 in each group) will be sufficient to provide

For peer review only - http://bmjopen.bmj.com/site/about/guidelines.xhtml

estimates of recruitment, retention, adherence and assessment of contamination within GP surgeries and between groups. This is broadly in-line with published "rules of thumb".[38 39]

Data management

Data will be entered onto the study database. The system will incorporate data entry and validation rules to reduce data entry errors, and management functions to facilitate auditing and data quality assurance.

Statistical methods

The aim will be to determine the feasibility of undertaking the main trial and explore acceptability. We will report our findings following the pilot and feasibility extension of the CONSORT guidance (2010), including a CONSORT diagram, descriptive and summary statistics, along with all important harms or unintended effects in each group.

Descriptive statistics will be used to compare recruitment, retention, adherence and contamination rates overall and between the two groups; and in the intervention group, test results and adherence to dietary advice. Completion rates, average score and distributions (as appropriate) will be reported for the proposed outcomes in the main trial, e.g. POEM and EASI.

Economic scoping

We will gather data on key costs and outcomes to assess the feasibility of carrying out a cost-effectiveness analysis from the primary perspective of the NHS and from a wider perspective including parental costs and time off work.

Data on healthcare contacts and prescribed medications will be extracted from EMRs. Additional healthcare contacts, information about parental out-of-pocket expenses and time off work will be collected using four-weekly parent-completed questionnaires. The overall level of missing data will be recorded and the pattern of missing data, by item, will be explored. Relevant unit costs will be identified and, once resource-use has been costed, we will identify which items are important cost drivers. The resources required for the intervention will be identified and the feasibility of costing these established.

NICE recommends the use of Quality-adjusted life-years (QALYs) as the preferred outcome measure in economic evaluations, but it is unclear what the most appropriate underlying measure is for this population in estimating QALYs. Therefore, we will test feasibility and validity of using both condition-specific (ADQoL)[32] and generic (CHU-9D) [35 36] preference-based health-related quality of life measures in children (measured at baseline, eight and 24 weeks) to estimate QALYs. The CHU-9D is currently validated for children aged 6 and over, with pilot versions for those aged 5-7 and additional guidance notes and validation questions for those under 5. One key component of the economic work will be to determine the feasibility of using the CHU-9D in this pre-school age group.

Nested qualitative study

The aims of the qualitative study are to help interpret and explain the quantitative feasibility findings (including experience and acceptability of study processes/intervention); and to generate new knowledge around the issues of food allergy, allergy tests and dietary modification in children with eczema, from the perspective of parents and GPs.

GPs at participating surgeries will be asked to complete a brief questionnaire and all parents and GPs will be asked whether they are willing to be contacted to take part in an interview. Semi-structured qualitative interviews will be conducted with a sample of trial parents and GPs from participating

For peer review only - http://bmjopen.bmj.com/site/about/guidelines.xhtml

surgeries, using topic guides developed based on study aims and input from the Trial Management Group (TMG).

Parents will be selected purposively to ensure diversity in relevant characteristics: trial group (intervention or comparator) with oversampling of the intervention group; eczema severity according to POEM (mild/moderate (<17) vs severe (≥17)); socio-economic status (assessed via postcode, using the Index of Multiple Deprivation Decile (categories: high (8-10)/medium (5-7)/low (1-4));[40] for mothers, whether currently breastfeeding; and length of time in the trial (shortly after baseline visit or OFC, or later in the trial). GPs will be purposively sampled to capture variation in GP surgery deprivation decile,[40] length of time in the trial, number of years' experience as a GP and confidence in managing children with eczema (assessed via a single item scored 1 (low) – 10 (high)). Sampling will stop when we have sufficient information power relevant to the study aims;[41] we anticipate a total of 20 parent and 10 GP interviews.

In addition, we will conduct brief telephone interviews with 5-8 parents who are ineligible to participate, decline to take part, or withdraw during the trial but indicate that they are willing to discuss reasons why. This information may provide valuable data to inform the design of a future definitive trial.

Interviews will be conducted by an experienced qualitative researcher, either by telephone or face-to-face, depending on the preference of the interviewee, audio-recorded (with permission) and transcribed verbatim. All interviewees will receive an information sheet and consent form to read in advance of the interview. Written informed consent will be taken prior to face-to-face interviews, and verbal consent will be taken for telephone interviews.

Data analysis of interview transcripts will take place alongside data collection and inform further data collection. We will conduct a thematic analysis, using both inductive and deductive coding (informed by the Common Sense model).[42]

Monitoring, safety and audit

Because this is a low-risk feasibility trial, the trial is over seen by a joint Trial Steering/Data Monitoring Committee (TS/DM-C) which is comprised of four independent members: a chairperson, a biostatistician, a clinician, and a patient representative (parent of child with eczema). Their role will be to provide overall supervision of the trial on behalf of the funder, with a focus on progress of the trial, adherence to the protocol, patient safety and consideration of new information.

Adverse events will be collected in the CRFs and by parent/clinician report and reported to the TMG and TS/DM-C. Possible serious adverse events include:

- severe localised reaction (redness, swelling, itch) to one or more SPTs necessitating medication and/or hospitalisation; and/or
- anaphylactic reaction (generalised flushing of the skin, hives, swelling of throat and mouth, difficulty in swallowing or speaking, tachycardia, severe asthma, abdominal pain and/or nausea and vomiting, hypotension and/or collapse and unconsciousness) requiring medication +/- hospitalisation (SPTs or OFC).

The sponsor organisation is the University of Bristol.

For peer review only - http://bmjopen.bmj.com/site/about/guidelines.xhtml

PRE-GRANT APPLICATION SURVEY

An online survey of parents of children with eczema informed the study design. It was promoted via social media and partner eczema and allergy websites between 10-27 October 2016. We received 152 responses, 97% (145/150) female with a mean age of 38.8 years. The median number of children with eczema was 1 (interquartile range 1,2) and the mean POEM score (for the worst affected child, where more than one child with eczema) was 11.7 (SD 7.6). 74% (108/146) had one or more food allergies, the most common being peanut, egg and cow's milk. 71.3% (77/108) had received allergy tests and been given advice by a healthcare professional and 17.6% (19/108) based their report on their observation of symptoms/reaction alone.

Participants were asked "In a study that compares the effect of doing allergy tests or giving advice on avoiding certain foods in children, what would be the single most important thing that this kind of study could tell you about?" Overall, 37% (56/151) chose "Reduce the risk of a sudden or severe allergic reaction". However, among those children without a reported food allergy (the group of interest in this study), 44% (16/36) chose "Reduce day-to-day severity of eczema". Consequently, we included eczema severity as a key clinical outcome.

Regarding the then proposed study, 96% (144/150) said they would be willing for their child to have an allergy test, with 67.1% (100/149) identifying skin prick as their "first choice" option for testing for allergy, and 54.3% (82/151) saying a blood test was an acceptable "second choice". Other participants said they would refuse (4.0% skin prick, 8.5% blood test) or did not know (2.0% skin prick, 2.6% blood test). Further information about the limitations of both types of test (risk of false reassurance or worry) did not change the opinion of the majority (72.5%, 108/149) of respondents. 56.9% (74/130) said that based on the clinical history and allergy test, they would be willing to avoid that food for at least 24 months. These findings provided reassurance as to the acceptability of the intervention, which includes skin prick tests and the possibility of having to exclude foods for at least several months.

PUBLIC AND PATIENT INVOLVEMENT

The James Lind Alliance eczema research priority setting partnership (2013) identified the following questions: "What role might food allergy tests play in treating eczema?" and "What is the role of [exclusion] diets in treating eczema?",[43] which a follow-on definitive trial could begin to address.

Two mothers of children with eczema (Gray & McMeechan) are members of the TMG and regularly attend the meetings. They have commented on the research proposal and study paperwork, and their suggestions around nomenclature and reducing data burden on participants have been incorporated. A lay member also sits on TS/DM-C.

We have established and met with a wider PPI advisory group. It first met towards the beginning of the research to discuss data burden and the design of patient facing materials. At a subsequent meeting, study progress and challenges were discussed. One more meeting is planned towards the end of the study, to inform write-up and dissemination of findings.

ETHICS AND DISSEMINATION

Research ethics approval

The study has been reviewed by the Health Research Authority and given a favourable opinion by the NHS REC (West Midlands – South Birmingham Research Ethics Committee, Reference Number 18/WM/0124).

For peer review only - http://bmjopen.bmj.com/site/about/guidelines.xhtml

Protocol amendments

Any amendments to the Protocol will be reported accordingly to the regulatory bodies, with a full copy of the current protocol available for download from the study website. Amendments to date are listed in appendix 1.

Consent or assent

Written consent for taking part in the trial will be received by a CSO from the parent or guardian of the participant at their baseline appointment, which takes place in a participating GP practice. Consent is also sought to contact participants regarding possible interview in the nested qualitative study; and for the re-use of the anonymised data in future research for purposes not related to this study, including as publicly available "open data". Consent for oral food challenges is received by the hospital nurse undertaking the procedure.

Confidentiality and access to data

The database and randomisation system will protect patient information in line with the data protection legislation. Trial staff will ensure that participants' anonymity is maintained through protective and secure handling and storage of patient information at the trial centre.

The Chief Investigator (CI) will have access to and act as custodian of the full dataset, which will be made available to the TS/DM-C if requested to verify the validity of the findings.

Ancillary and post-trial care

Participants requiring follow-up beyond their six months in the study will be referred by their GP to their local allergy clinic.

Dissemination and data sharing

Study progress, outputs and a summary of findings will be made available via a study website and Twitter account; and summaries distributed to participating families and GP surgeries. Findings will be submitted for presentation at conferences and written up for publication in a peer-reviewed journal(s), which may include mixed-method triangulation and integration of the quantitative and qualitative findings.

No later than three years after the completion of the study, we will make available a completely deidentified data set to an appropriate data archive for sharing purposes.

DISCUSSION

There are wide variations in provision of allergy testing for children with eczema. Parental concern and clinician uncertainty about the role of food allergy in eczema has been highlighted as a barrier to effective treatment.[44] Up to 70% of parents make significant modifications to their child's diet, often without professional advice,[45] even if the child has only mild eczema. Many parents turn to the internet for advice,[12 46] or purchase self-test allergy kits which are not validated and not recommended.[16]

It is uncommon for allergy tests to be undertaken in primary care but in principle, allergy testing (in the form of skin prick tests) and advice could be routinely delivered in primary care, but evidence is required to demonstrate both the feasibility and value of doing so. An RCT is needed to determine the clinical and cost-effectiveness of food allergy testing and advice in primary care, on severity of eczema in children. There are potentially significant benefits for the NHS of improving long-term eczema management, avoiding serious allergic reactions, and targeting child nutrition. This study

For peer review only - http://bmjopen.bmj.com/site/about/guidelines.xhtml

will provide important data to first, determine the feasibility of a large, definitive trial; and second, to inform its design.

The full/most up-to-date version of the protocol is available to download from the study website. The first participant was randomised in September 2018 and recruitment is on-going. Follow-up is expected to be complete by September 2019. We expect to report in early 2020.

STATEMENTS

Acknowledgements

This study was designed and is being delivered in collaboration with the Bristol Randomised Trials Collaboration (BRTC), a UKCRC registered clinical trials unit which, as part of the Bristol Trials Centre, is in receipt of National Institute for Health Research CTU support funding.

The study was developed with support from UK Dermatology Clinical Trials Network (UK DCTN). The UK DCTN is grateful to the British Association of Dermatologists and the University of Nottingham for financial support of the Network.

We are grateful to the Centre for Evidence Based Dermatology (www.nottingham.ac.uk/research/groups/cebd), Nottingham Eczema Support Group for Carers of Children with Eczema (www.nottinghameczema.org.uk/), the National Eczema Society (www.eczema.org) and Allergy UK (www.allergyuk.org), Anaphylaxis Campaign (www.anaphylaxis.org.uk) for their helping in promoting the pre-grant PPI survey.

Trial steering and data monitoring committee members: Professor Carl Heneghan (chair), Dr David Gillespie (statistician), Dr Joanne Walsh (GP with specialist interest in allergy) and Kate Sykes (parent of child with eczema and food allergy).

Declaration of interests

MR: No financial interests; convenes the NIHR SPCR Allergy working group; and was a member of the NICE Quality Standard 44 for Atopic eczema in under 12s and RCPCH “Care pathway for children with eczema” groups.

LW: Direct – financial: write articles, attend round table infant formula company meetings and present at sponsored lectures relating to food allergy; received infant formula company sponsorship to attend national/international allergy related conferences/ meetings. Runs a private practice (Food Allergy Nottingham Service Ltd, 2013-) in addition to my NHS role (Feb 2012-). BDA cow’s milk allergy course facilitator (2018-). Direct – non-financial: member of RCPCH faculty for tier 3 paediatric allergy course (2018-); member of Allergy UK health advisory board (2015-); member of iMAP implementation team (2017-); produce food allergy related dietary information for BDA food allergy group (2014-); NICE Expert adviser relating to paediatric food allergy and gastro-oesophageal reflux (2017-2020); previous member of NICE food allergy guidelines GDG and RCPCH food allergy care pathway (2010-2011)

RJB: RJB has received honoraria for participating in advisory boards for ALK-Abello who manufacture allergy diagnostics and treatments, and DBV technologies who manufacture a food allergy treatment.

For peer review only - http://bmjopen.bmj.com/site/about/guidelines.xhtml

Funding

The study is funded by National Institute for Health Research (NIHR) School for Primary Care Research (project 383). MR is funded by an NIHR Post-Doctoral Research Fellowship (PDF-2014-07-013). The views expressed in this publication are those of the authors and not necessarily those of the NHS, the National Institute for Health Research or the Department of Health and Social Care.

Contributorship statement

MR conceived the study idea in collaboration with RB, MS, JCh, IM and LE; MR, RB, MS, JCh, IM, LE, AH, DM, KR, KG, PB and JK developed the initial study design, with later input from JCo, LS, LW, EA and JT. Specific advice was given by PB and NT on trial design and medical statistics; AH, LS and CC on the nested qualitative study; and KG and JCo on the economic scoping. All the authors contributed to the drafting of the study protocol, led by MR, and approved the final manuscript.

The TMG would like to thank the early input of Professor Hywel Williams (Centre for Evidence Based Dermatology, University of Nottingham), Dr Sandra Hollinghurst and Dr Jeremy Horwood (University of Bristol) and Dr Kate Grimshaw (University of Southampton) for their advice on study design and delivery.

Study website and social media

www.bristol.ac.uk/eczema-allergy-study and Twitter: @eczema-allergy

REFERENCES

1. Johansson S, Bieber T, Dahl R, et al. Revised nomenclature for allergy for global use: Report of the Nomenclature Review Committee of the World Allergy Organization, October 2003. *J Allergy Clin Immunol* 2004; 113: 832-36.
2. Eichenfield LF, Tom WL, Chamlin SL, et al. Guidelines of care for the management of atopic dermatitis: Part 1. Diagnosis and assessment of atopic dermatitis. *Journal of the American Academy of Dermatology* 2014; 70(2): 338-51. doi: 10.1016/j.jaad.2013.10.010
3. Santer M, Burgess H, Yardley L, et al. Experiences of carers managing childhood eczema and their views on its treatment: a qualitative study. *British Journal of General Practice* 2012; 62(597): e261-e67. doi: 10.3399/bjgp12X636083
4. Santer M, Burgess H, Yardley L, et al. Managing childhood eczema: qualitative study exploring carers' experiences of barriers and facilitators to treatment adherence. *Journal of Advanced Nursing* 2013; 69(11): 2493-501. doi: 10.1111/jan.12133
5. Powell K, Le Roux E, Banks J, et al. GP and parent dissonance about the assessment and treatment of childhood eczema in primary care: a qualitative study. *BMJ Open* 2018; 8(2) doi: 10.1136/bmjopen-2017-019633
6. Martin PE, Eckert JK, Koplin JJ, et al. Which infants with eczema are at risk of food allergy? Results from a population-based cohort. *Clinical & Experimental Allergy* 2015; 45(1): 255-64. doi: 10.1111/cea.12406
7. Cox H, Lloyd K, Williams H, et al. Emollients, education and quality of life: the RCPCH care pathway for children with eczema. *Archives of Disease in Childhood* 2011; 96(Suppl 2): i19-i24.
8. Bath-Hextall F, Delamere FM, Williams HC. Dietary exclusions for improving established atopic eczema in adults and children: systematic review. *Allergy* 2009; 64(2): 258-64.
9. Lever R, MacDonald C, Waugh P, et al. Randomised controlled trial of advice on an egg exclusion diet in young children with atopic eczema and sensitivity to eggs. *Pediatric Allergy and Immunology* 1998; 9(1): 13-19. doi: 10.1111/j.1399-3038.1998.tb00294.x

For peer review only - http://bmjopen.bmj.com/site/about/guidelines.xhtml

10. Chafen J, Newberry SJ, Riedl MA, et al. Diagnosing and managing common food allergies: A systematic review. *JAMA* 2010; 303(18): 1848-56. doi: 10.1001/jama.2010.582
11. Nankervis H, Thomas K, Delamere F, et al. Scoping systematic review of treatments for eczema. *Programme Grants Appl Res* 2016;4(7)
12. Halls A, Nunes D, Muller I, et al. 'Hope you find your 'eureka' moment soon': a qualitative study of parents/carers' online discussions around allergy, allergy tests and eczema. *BMJ Open* 2018; 8(11) doi: 10.1136/bmjopen-2018-022861
13. Eldridge SM, Lancaster GA, Campbell MJ, et al. Defining Feasibility and Pilot Studies in Preparation for Randomised Controlled Trials: Development of a Conceptual Framework. *PLoS ONE* 2016; 11(3): e0150205. doi: 10.1371/journal.pone.0150205
14. Charman C, Venn A, Ravenscroft J, et al. Translating Patient-Oriented Eczema Measure (POEM) scores into clinical practice by suggesting severity strata derived using anchor-based methods. *Br J Dermatol* 2013; 169(6): 1326-32. doi: 10.1111/bjd.12590
15. RCPCH Allergy Care Pathways Project. Taking an allergy focused clinical history. https://www.rcpch.ac.uk/sites/default/files/Taking_an_Allergy_Focused_Clinical_History_-_Allergy_Care_Pathways_Project.pdf [Accessed 07.12.18]
16. National Institute for Health and Clinical Excellence. Food allergy in children and young people - Diagnosis and assessment of food allergy in children and young people in primary care and community settings. London: NICE, 2011.
17. Chalmers JR, Haines RH, Mitchell EJ, et al. Effectiveness and cost effectiveness of daily all over body application of emollient during the first year of life for preventing atopic eczema in high risk children (The BEEP trial): study protocol for a randomised controlled trial. *Trials* 18: 343 DOI : 10.1186/s13063-017-2031-3

18. Burks AW, James JM, Hiegel A, et al. Atopic dermatitis and food hypersensitivity reactions. *The Journal of Pediatrics* 1998;132(1):132-36. doi: http://dx.doi.org/10.1016/S0022-3476(98)70498-6

19. Niggemann, Sielaff, Beyer, et al. Outcome of double-blind, placebo-controlled food challenge tests in 107 children with atopic dermatitis. *Clinical & Experimental Allergy* 1999; 29(1): 91-96. doi: 10.1046/j.1365-2222.1999.00454.x
20. Heinzerling L, Mari A, Bergmann K-C, et al. The skin prick test – European standards. *Clinical and Translational Allergy* 2013; 3(1): 3. doi: 10.1186/2045-7022-3-3
21. Sampson HA, van Wijk RG, Bindslev-Jensen C, et al. Standardizing double-blind, placebo-controlled oral foodchallenges: American Academy of Allergy, Asthma & Immunology–European Academy of Allergy and Clinical Immunology PRACTALL consensus report. *J Allergy Clin Immunol* 2012; 130 doi: 10.1016/j.jaci.2012.10.017
22. Ricci G, Dondi A, Patrizi A. Useful tools for the management of atopic dermatitis. *Am J Clin Dermatol* 2009; 10: 287-300.
23. Grabenhenrich LB, Reich A, Bellach J, et al. A new framework for the documentation and interpretation of oral food challenges in population-based and clinical research. *Allergy* 2017; 72(3): 453-61. doi: 10.1111/all.13049
24. National Institute for Health and Clinical Excellence. CG057: Atopic eczema in children - management of atopic eczema in children from birth up to the age of 12 years. London: RCOG Press, 2007.
25. Schmitt J, Spuls P, Boers M, et al. Towards global consensus on outcome measures for atopic eczema research: results of the HOME II meeting. *Allergy* 2012; 67: 1111-17. doi: 10.1111/j.1398-9995.2012.02874.x
26. Chalmers JR, Simpson E, Apfelbacher CJ, et al. Report from the fourth international consensus meeting to harmonize core outcome measures for atopic eczema/dermatitis

For peer review only - http://bmjopen.bmj.com/site/about/guidelines.xhtml

clinical trials (HOME initiative). *British Journal of Dermatology* 2016 Jul; 175(1): 69-79 doi: 10.1111/bjd.14773

27. Charman CR, Venn AJ, Williams HC. The Patient-Oriented Eczema Measure: Development and Initial Validation of a New Tool for Measuring Atopic Eczema Severity From the Patients' Perspective. *Archives of Dermatology* 2004; 140(12): 1513-19.
28. Thomas K, Bradshaw L, Montgommery A, et al. Using existing trial data to inform the development of core outcome sets and improve efficiencies in research. *Trials* 2015; 16(Suppl 2): O74.
29. Schmitt J, Langan S, Williams HC. What are the best outcome measurements for atopic eczema? A systematic review. *Journal of Allergy and Clinical Immunology* 2007; 120(6): 1389-98. doi: 10.1016/j.jaci.2007.08.011
30. Gaunt DM, Metcalfe C, Ridd M. The Patient-Oriented Eczema Measure in young children: responsiveness and minimal clinically important difference *Allergy* 2016; 71: 1620–1625 doi: 10.1111/all.12942
31. Hanifin JM, Thurston M, Omoto M, et al. The eczema area and severity index (EASI): assessment of reliability in atopic dermatitis. *Experimental Dermatology* 2001; 10(1): 11-18.
32. Stevens KJ, Brazier JE, McKenna SP, et al. The development of a preference-based measure of health in children with atopic dermatitis. *British Journal of Dermatology* 2005; 153(2): 372-77.
33. Basra MKA, Gada V, Ungaro S, et al. Infants' Dermatitis Quality of Life Index: a decade of experience of validation and clinical application. *British Journal of Dermatology* 2013; 169(4): 760-68. doi: 10.1111/bjd.12563
34. Lewis-Jones MS, Finlay AY, Dykes PJ. The Infants Dermatitis Quality of Life Index. *British Journal of Dermatology* 2001; 144(1): 104-10.
35. Stevens K. Assessing the performance of a new generic measure of health-related quality of life for children and refining it for use in health state valuation. *Applied Health Economics and Health Policy* 2011; 9(3): 157-69.
36. Stevens K, Katherine S. Valuation of the Child Health Utility 9D Index. *Pharmacoeconomics* 2012; 30: 729-47.
37. Canaway AG, Frew EJJQoLR. Measuring preference-based quality of life in children aged 6–7 years: a comparison of the performance of the CHU-9D and EQ-5D-Y—the WAVES Pilot Study. 2013; 22(1): 173-83. doi: 10.1007/s11136-012-0119-5
38. Browne R. On the use of a pilot sample for sample-size determination. *Stat Med* 1995; 14(17): 1933 - 40.
39. Teare M, Dimairo M, Shephard N, et al. Sample size requirements to estimate key design parameters from external pilot randomised controlled trials: a simulation study. *Trials* 2014; 15(1): 264.
40. Smith T, Noble M, Noble S, et al. The English Indices of Deprivation 2015. London, 2015.
41. Malterud K, Siersma VD, Guassora AD. Sample Size in Qualitative Interview Studies: Guided by Information Power. *Qualitative Health Research* 2016; 26(13): 1753-60. doi: 10.1177/1049732315617444
42. Hagger MS, Orbell S. A Meta-Analytic Review of the Common-Sense Model of Illness Representations. *Psychology & Health* 2003; 18(2): 141-84. doi: 10.1080/088704403100081321
43. Batchelor JM, Ridd MJ, Clarke T, et al. The Eczema Priority Setting Partnership: a collaboration between patients, carers, clinicians and researchers to identify and prioritize important research questions for the treatment of eczema. *British Journal of Dermatology* 2013; 168(3): 577-82. doi: 10.1111/bjd.12040
44. Le Roux E, Powell K, Banks JP, et al. GPs' experiences of diagnosing and managing childhood eczema: a qualitative study in primary care. *British Journal of General Practice* 2018; 68(667): e73-e80. doi: 10.3399/bjgp18X694529

For peer review only - http://bmjopen.bmj.com/site/about/guidelines.xhtml

45. Chan JP, Ridd MJ. Carer and patient beliefs and practices regarding the role of food allergy in eczema: cross-sectional survey. *Clinical & Experimental Dermatology* In submission

46. Santer M, Muller I, Yardley L, et al. 'You don't know which bits to believe': qualitative study exploring carers' experiences of seeking information on the internet about childhood eczema. *BMJ Open* 2015;5(4) doi: 10.1136/bmjopen-2014-006339

47. Werfel T, Erdmann S, Fuchs T, et al. Approach to suspected food allergy in atopic dermatitis. *JDDG: Journal der Deutschen Dermatologischen Gesellschaft* 2009;7(3):265-71. doi: 10.1111/j.1610-0387.2008.06901.x

For peer review only

For peer review only - http://bmjopen.bmj.com/site/about/guidelines.xhtml

TABLES

Table 1: IgE and non-IgE mediated food allergy

- The World Allergy Organisation defines food allergy as an immune-mediated hypersensitivity reaction to food and may be divided into Immunoglobulin E (IgE) mediated and non-IgE mediated reactions.[1]
- IgE-mediated food allergy involves immediate hypersensitivity (typically within 5-30 minutes of ingestion and always within 2 hours) through the action of mast cells. It can be reliably diagnosed when there is a typical history of reaction within 1-2 hours of exposure and demonstration of specific IgE to the relevant food on blood or skin prick testing.
- Non-IgE mediated food allergy is delayed (between 2-48 hours post ingestion) and thought to be caused by an aberrant T-cell response. It is more difficult to diagnose as there are no reliable diagnostic tests other than dietary exclusions and re-introduction.[47]

Table 2: Participant eligibility criteria

Inclusion criteria are:

- child aged between 3 months and 5 years with eczema diagnosed by an appropriately qualified healthcare professional (registered doctor, nurse or health visitor)
- Patient Orientated Eczema Measure (POEM) score of >2
- consent given by a person with parental responsibility for the participant

Exclusion criteria are:

- child with medically-diagnosed food allergy, awaiting referral/investigations for possible food allergy, or had previous investigations for food allergy (not including home testing)
- the person responsible for consent has insufficient written English to complete the outcome measures, or has another child already taking part in the trial.

For peer review only - http://bmjopen.bmj.com/site/about/guidelines.xhtml

Table 3: Schedule of enrolment, interventions and assessments

	Study period									
	Enrolment	Allocation	Post-allocation							Close-out
		V_0	Follow-up questionnaires						V_1	
Week		0	4	8	12	16	20	24	24	
Parent-completed										
Screening questionnaire	●									
Demographics and medical history		●								
POEM	●	●	●	●	●	●	●	●		
Other eczema symptoms†		●	●	●	●	●	●	●		
Other possible symptoms of food allergy				●				●		
Diet of child (and breast-feeding mother)			●	●	●	●	●	●		
Health service utilisation			●	●	●	●	●	●		
Out-of-pocket expenses/time off work			●	●	●	●	●	●		
ADQoL		●		●				●		
CHU-9D		●		●				●		
IDQoL		●		●				●		
Parental anxiety (GAD-7)		●						●		
Exit questionnaire								●		
Researcher-administered										
UK Diagnostic criteria for atopic dermatitis		●								
Other possible symptoms of food allergy		●								
Diet of child (and breast-feeding mother)		●								
EASI		●							●	
Structured allergy history		○								
Skin Prick Test (SPT)		○								
Oral Food Challenge (OFC)		*								
Home dietary trial		*								
EMR notes review										●

V_0 = baseline visit; V_1 = follow-up visit at 24weeks

● All participants; ○ only participants in intervention group; * only participants in intervention group with equivocal structured allergy history/SPT results

POEM: Patient Orientated Eczema Measure; † bother score, itch intensity, parent global assessment; ADQoL: Atopic Dermatitis Quality of Life; IDQoL: Infant Dermatitis Quality of Life; CHU-9D: Children's Health Utility 9D; GAD-7: Generalised Anxiety Disorder 7; EASI: Eczema Area Severity Index.

For peer review only

For peer review only - http://bmjopen.bmj.com/site/about/guidelines.xhtml

For peer review only

For peer review only - http://bmjopen.bmj.com/site/about/guidelines.xhtml

Figure 1: Overview of participant pathway through the study

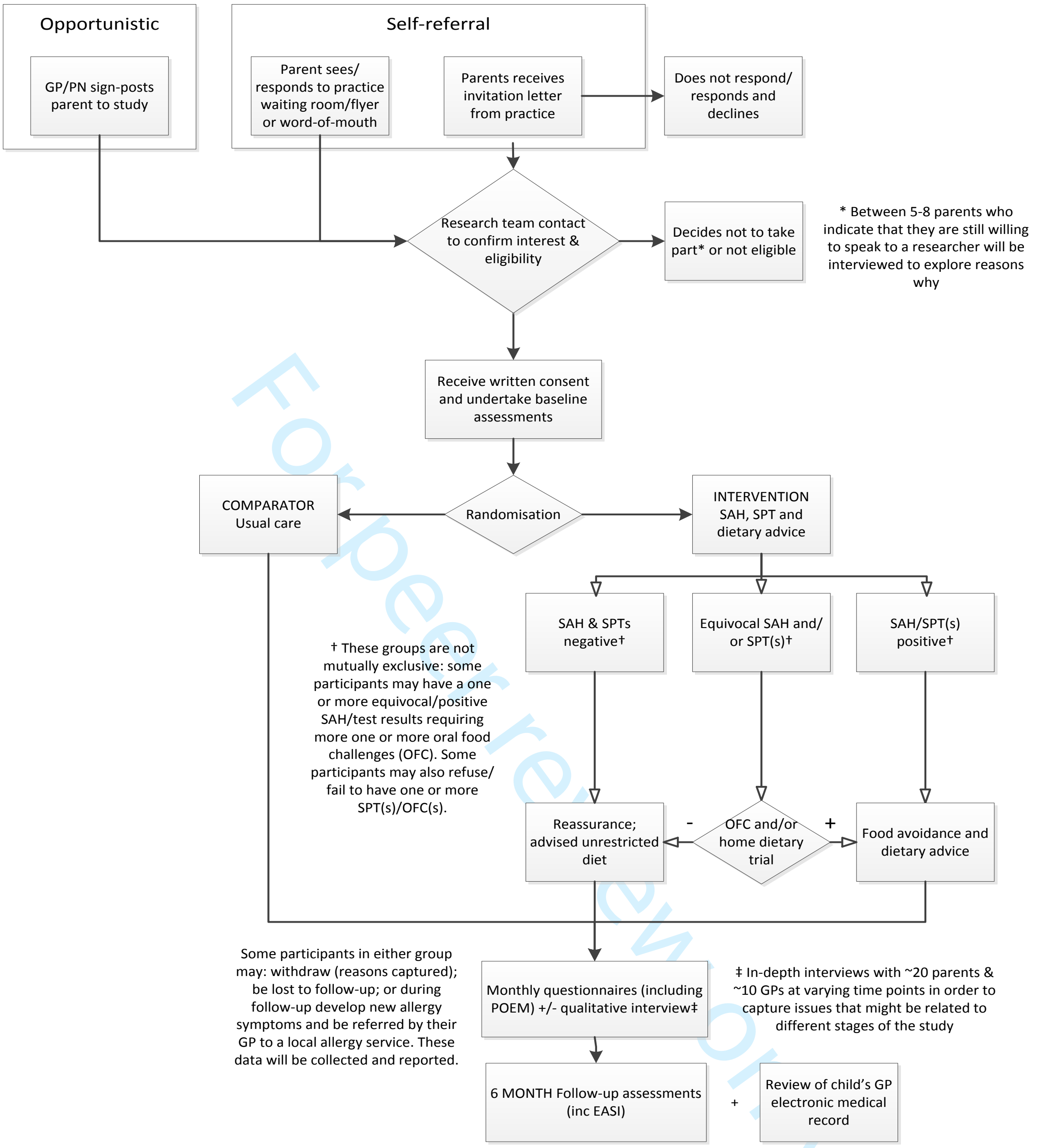

+

For peer review only

Appendix 1: Administrative information

Title

TEST (Trial of Eczema allergy Screening Tests): a single centre, individually randomised, two-group feasibility randomised controlled trial of allergy tests in children with eczema, with economic scoping and nested qualitative study

Trial registration number

ISRCTN: 15397185 (30 July 2018)

World Health Organization Trial Registration Data Set

Data category	Information
Primary registry and trial identifying number	ISRCTN 15397185
Date of registration in primary registry	30 July 2018
Secondary identifying numbers	IRAS: 237046<br>NHS REC: 18/WM/0124
Source(s) of monetary or material support	NIHR School for Primary Care Research
Primary sponsor	University of Bristol
Secondary sponsor(s)	Not applicable
Contact for public queries	Mr Doug Webb, test-study@bristol.ac.uk, 0117 928 7351
Contact for scientific queries	Dr Matthew Ridd FRCGP PhD, m.ridd@bristol.ac.uk, 0117 331 4557
Public title	Trial of Eczema allergy Screening Tests (TEST)
Scientific title	The TEST (Trial of Eczema Allergy Screening Tests) study: feasibility randomised controlled trial with economic scoping and nested qualitative study
Countries of recruitment	England
Health condition(s) or problem(s) studied	Childhood eczema
Intervention(s)	Structured allergy history and skin prick tests; depending on outcome of these tests, reassurance, repeat skin prick test(s), oral food challenge and/or home dietary trial of exclusion or inclusion
Key inclusion and exclusion criteria	Inclusion: aged between 3 months and less than 5 years; eczema diagnosed by an appropriately qualified healthcare professional; mild, moderate or severe eczema (Patient Orientated Eczema Measure (POEM) score>2)<br>Exclusion: medically-diagnosed food allergy or awaiting referral/investigations for possible food allergy; previous investigations for food allergy (does not include home testing)
Study type	Intervention

For peer review only

Date of first enrolment	12 September 2018
Target sample size	80
Recruitment status	Recruiting
Primary outcome(s)	The feasibility of conducting the trial (recruitment, retention, contamination) and collecting the required data: recruitment and retention rates; acceptability of recruitment, intervention and follow-up procedures to parents/carers; acceptability of trial processes and procedures to GPs; development and refinement of a manual on the interpretation of test results and dietary advice to be given; number of participants In the intervention group with positive/negative tests; adherence to dietary advice; contamination of the control group; acceptability and feasibility of collecting clinical outcomes; feasibility and optimise collection of patient-level data on NHS and personal resource use; feasibility of using the CHU-9D in children under 5 years of age; inform eligibility criteria for the future definitive trial; detection bias in the collection of patient-reported outcomes; trial processes and logistics
Key secondary outcomes	Eczema symptoms, measured using POEM; eczema signs, measured using EASI; eczema 'bother' score; itch intensity score; parent global assessment of eczema; other possible symptoms of food allergy; UK diagnostic criteria for atopic dermatitis; main carer anxiety, measured using GAD-7; diet of child and/or mother if child being breastfed by her; adverse events; child and family quality of life, measured using ADQoL, CHU-9D and IDQoL; satisfaction with trial processes, procedures and paperwork; health services utilisation; out-of-pocket expenses/time off work.

Protocol version

Version 2.0 (18 October 2018)

**Version**		**Notes**
**Number**	**Date**	
2.0	18.10.18	Section 5.2: addition of missing data collection points (Diet of child and breast-feeding mother at baseline; ADQoL at 8 weeks)<br><br>Section 8.4: change "avoidance of food(s) with dietary advice; and referral via GP for follow-up" to "avoidance of food(s) with dietary advice; and referral to the local NHS allergy services via GP for longer-term follow-up" ; and "Any participants with indeterminate results will be reviewed by an expert allergy panel (co-applicants Boyle & Marriage) …" to "All participants' results will be reviewed by an expert allergy panel (including co-applicants Ridd, Boyle, Marriage and/or Waddell) …"<br><br>Section 9.1: change "Up to 12 GP surgeries" to "At least 12 GP surgeries …";<br><br>Section 9.3 & section 10.2: change "in/at their [own] GP practice" to "at a participating GP practice";<br><br>Section 10.1: description of expression of interest form corrected from "The form will comprise which will comprise POEM, questions asking their opinion of the role of diet/food allergy in their child's eczema, and any

For peer review only

		previous food allergy tests, diagnoses and/or dietary modifications" to "The form asks if they currently have eczema/a medically diagnosed food allergy and the POEM questions"<br><br>Section 11.3: Change from "In addition, we will conduct brief telephone interviews with ~5-8 parents who decline to take part in response to the initial invitation letter or later withdrawal from the trial but indicate that they are willing to discuss reasons why" to "In addition, we will conduct brief telephone interviews with ~5-8 parents who are ineligible, decline to take part or withdrawal from the trial but indicate that they are willing to discuss reasons why"<br><br>Section 16.1: change "Expected SAEs defined in the study protocol (page 39) …" to "Expected SAEs as defined below …"<br><br>Section 18.1: revised project duration/milestones<br><br>Other minor changes (correction of typing errors, changes in research team)
1.0	29.03.18	Submitted/approved by REC/HRA

Funding

NIHR School for Primary Care Research

Contributorship

See main manuscript

Sponsor contact information

Trial sponsor: University of Bristol
Sponsor's reference: 2832
Contact name: Mrs Anna Brooke
Address: Research Enterprise Development, One Cathedral Square, Bristol BS1 5DD
Email: research-governance@bristol.ac.uk
Telephone: 0117 428 4011

Role of study sponsor and funder

The funder and sponsor had no role in the design of this study and will not have any role during its execution, analyses, interpretation of the data, or decision to submit results.

Committees

The Trial Management Group (TMG) comprises all investigators, the trial manager, research and administrative staff, with input from patient/public representatives. Members of the TMG will contribute to the trial in the following ways: trial design and methods; participant recruitment and trial conduct; trial management; trial logistics and cost management; economic evaluation; qualitative study statistical data analysis; and publication. The TMG will meet on a regular basis to oversee the management of the trial. The TMG will be provided with detailed information by the centre staff regarding trial progress. Meetings will be face-to-face with teleconference facilities for TMG members who are unable to be present.

For peer review only

This study was designed and is being delivered in collaboration with the Bristol Randomised Trials Collaboration (BRTC), a UKCRC registered clinical trials unit which, as part of the Bristol Trials Centre, is in receipt of National Institute for Health Research CTU support funding. Members of the BRTC will attend the TMG.

Because this is a low-risk trial, the funder has agreed that the roles of both guiding the Trial Management Group and monitoring trial data will be undertaken by a single Trial Steering/Data Monitoring Committee (TS/DM-C). The TS/DM-C will meet at least three times over the course of the study and comprises four independent members: a chairperson, a biostatistician, a clinician, and a patient representative (parent of child with eczema). Their role will be to provide overall supervision of the trial on behalf of the funder, with a focus on progress of the trial, adherence to the protocol, patient safety and consideration of new information. The committee will review the accruing data and assess whether there are any safety issues that should be brought to the Sponsor's or the participants' attention or any reasons for the trial not to continue. Terms of reference will be drawn up and agreed with members of the TS/DM-C.

For peer review only

University of Bristol · University of Southampton · The University of Nottingham (United Kingdom · China · Malaysia) · Bristol Randomised Trials Collaboration

The Trial of Eczema allergy Screening Tests (TEST) Study

Parent/Carer Consent Form

Initial box

1. I confirm that I have read and understand the Participant Information Sheet dated 18.10.18 Version 2.0 for the above study. I have had the opportunity to consider the information, ask questions and have had these questions answered satisfactorily. ☐
2. I understand that participation is voluntary and that we are free to withdraw at any time without giving any reason, without my child's medical care or legal rights being affected. ☐
3. I understand that after the study ends, the data collected will be made "open data". I understand that this means the anonymised data will be publicly available and may be used for purposes not related to this study. I understand that it will not be possible to identify me from these data. ☐
4. I understand that relevant sections of my child's medical notes and all information collected for this research may be reviewed by the study team, by the participating NHS Trust to ensure that the research is conducted appropriately. I give permission for these individuals to access my child's records as appropriate. ☐
5. I agree that my child's family doctor (GP) will be told that they are taking part in the study ☐
6. I give consent for the data collected in this trial to be used in future ethically approved studies on the understanding that all information will continue to be kept securely and remain confidential. ☐
7. I give consent to be contacted by a member of the research team with a view to being interviewed about my opinions about allergy testing for eczema and taking part in TEST. I understand that if I am contacted, I will be given more information about the interviews by the research team, I can decide later about taking part and I understand I will be asked to give further consent. ☐
8. I agree for myself and my child to take part in the above-named study. ☐

Name of Participant (Child)	Participant ID	
Name of Parent/Guardian	Signature	Date

[illegible]mpleted: 1 (original) for research team, 1 for participant

Funded by NHS National Institute for Health Research

The research was funded by the NIHR Health Technology Assessment (HTA) Programme. The views expressed are those of the author(s) and not necessarily those of the NHS, the NIHR or the Department of Health.

For peer review only - http://bmjopen.bmj.com/site/about/guidelines.xhtml

For peer review only

University of BRISTOL · UNIVERSITY OF Southampton · The University of Nottingham UNITED KINGDOM · CHINA · MALAYSIA · Bristol Randomised Trials Collaboration

Name of person receiving consent	Signature	Date

For peer review only

[illegible]mpleted: 1 (original) for research team, 1 for participant

Funded by NHS National Institute for Health Research
The research was funded by the NIHR Health Technology Assessment (HTA) Programme. The views expressed are those of the author(s) and not necessarily those of the NHS, the NIHR or the Department of Health.

For peer review only - http://bmjopen.bmj.com/site/about/guidelines.xhtml

SPIRIT

STANDARD PROTOCOL ITEMS: RECOMMENDATIONS FOR INTERVENTIONAL TRIALS

SPIRIT 2013 Checklist: Recommended items to address in a clinical trial protocol and related documents*

Section/item	ItemNo	Description	Location
**Administrative information**			
Title	1	Descriptive title identifying the study design, population, interventions, and, if applicable, trial acronym	Page 1
Trial registration	2a	Trial identifier and registry name. If not yet registered, name of intended registry	Page 2
	2b	All items from the World Health Organization Trial Registration Data Set	Appendix 1
Protocol version	3	Date and version identifier	Appendix 1
Funding	4	Sources and types of financial, material, and other support	Page 11
Roles and responsibilities	5a	Names, affiliations, and roles of protocol contributors	Page 11
	5b	Name and contact information for the trial sponsor	Page 8/Appendix 1
	5c	Role of study sponsor and funders, if any, in study design; collection, management, analysis, and interpretation of data; writing of the report; and the decision to submit the report for publication, including whether they will have ultimate authority over any of these activities	Page 11/Appendix 1
	5d	Composition, roles, and responsibilities of the coordinating centre, steering committee, endpoint adjudication committee, data management team, and other individuals or groups overseeing the trial, if applicable (see Item 21a for data monitoring committee)	Page 8
**Introduction**			

For peer review only

Background and rationale	6a	Description of research question and justification for undertaking the trial, including summary of relevant studies (published and unpublished) examining benefits and harms for each intervention	Page 3
	6b	Explanation for choice of comparators	Page 3
Objectives	7	Specific objectives or hypotheses	Pages 3-4
Trial design	8	Description of trial design including type of trial (eg, parallel group, crossover, factorial, single group), allocation ratio, and framework (eg, superiority, equivalence, noninferiority, exploratory)	Page 4
**Methods: Participants, interventions, and outcomes**			
Study setting	9	Description of study settings (eg, community clinic, academic hospital) and list of countries where data will be collected. Reference to where list of study sites can be obtained	Page 4
Eligibility criteria	10	Inclusion and exclusion criteria for participants. If applicable, eligibility criteria for study centres and individuals who will perform the interventions (eg, surgeons, psychotherapists)	Page 4/table 2
Interventions	11a	Interventions for each group with sufficient detail to allow replication, including how and when they will be administered	Pages 4-5
	11b	Criteria for discontinuing or modifying allocated interventions for a given trial participant (eg, drug dose change in response to harms, participant request, or improving/worsening disease)	N/A
	11c	Strategies to improve adherence to intervention protocols, and any procedures for monitoring adherence (eg, drug tablet return, laboratory tests)	Page 6
	11d	Relevant concomitant care and interventions that are permitted or prohibited during the trial	Page 5

For peer review only - http://bmjopen.bmj.com/site/about/guidelines.xhtml

Outcomes	12	Primary, secondary, and other outcomes, including the specific measurement variable (eg, systolic blood pressure), analysis metric (eg, change from baseline, final value, time to event), method of aggregation (eg, median, proportion), and time point for each outcome. Explanation of the clinical relevance of chosen efficacy and harm outcomes is strongly recommended	Pages 5-6, table 3
Participant timeline	13	Time schedule of enrolment, interventions (including any run-ins and washouts), assessments, and visits for participants. A schematic diagram is highly recommended (see Figure)	Pages 4 & 6, Figure 1 and Table 3
Sample size	14	Estimated number of participants needed to achieve study objectives and how it was determined, including clinical and statistical assumptions supporting any sample size calculations	Page 6
Recruitment	15	Strategies for achieving adequate participant enrolment to reach target sample size	Pages 4 & 6
**Methods: Assignment of interventions (for controlled trials)**			
Allocation:			
Sequence generation	16a	Method of generating the allocation sequence (eg, computer-generated random numbers), and list of any factors for stratification. To reduce predictability of a random sequence, details of any planned restriction (eg, blocking) should be provided in a separate document that is unavailable to those who enrol participants or assign interventions	Page 4
Allocation concealment mechanism	16b	Mechanism of implementing the allocation sequence (eg, central telephone; sequentially numbered, opaque, sealed envelopes), describing any steps to conceal the sequence until interventions are assigned	Page 4
Implementation	16c	Who will generate the allocation sequence, who will enrol participants, and who will assign participants to interventions	Page 4

For peer review only - http://bmjopen.bmj.com/site/about/guidelines.xhtml

Blinding (masking)	17a	Who will be blinded after assignment to interventions (eg, trial participants, care providers, outcome assessors, data analysts), and how	
	17b	If blinded, circumstances under which unblinding is permissible, and procedure for revealing a participant's allocated intervention during the trial	Page 6
**Methods: Data collection, management, and analysis**			
Data collection methods	18a	Plans for assessment and collection of outcome, baseline, and other trial data, including any related processes to promote data quality (eg, duplicate measurements, training of assessors) and a description of study instruments (eg, questionnaires, laboratory tests) along with their reliability and validity, if known. Reference to where data collection forms can be found, if not in the protocol	Page 6 and Table 3
	18b	Plans to promote participant retention and complete follow-up, including list of any outcome data to be collected for participants who discontinue or deviate from intervention protocols	Page 6
Data management	19	Plans for data entry, coding, security, and storage, including any related processes to promote data quality (eg, double data entry; range checks for data values). Reference to where details of data management procedures can be found, if not in the protocol	Page 6
Statistical methods	20a	Statistical methods for analysing primary and secondary outcomes. Reference to where other details of the statistical analysis plan can be found, if not in the protocol	Page 6
	20b	Methods for any additional analyses (eg, subgroup and adjusted analyses)	N/A
	20c	Definition of analysis population relating to protocol non-adherence (eg, as randomised analysis), and any statistical methods to handle missing data (eg, multiple imputation)	N/A
**Methods: Monitoring**			

Data monitoring	21a	Composition of data monitoring committee (DMC); summary of its role and reporting structure; statement of whether it is independent from the sponsor and competing interests; and reference to where further details about its charter can be found, if not in the protocol. Alternatively, an explanation of why a DMC is not needed	Page 8
	21b	Description of any interim analyses and stopping guidelines, including who will have access to these interim results and make the final decision to terminate the trial	Page 8
Harms	22	Plans for collecting, assessing, reporting, and managing solicited and spontaneously reported adverse events and other unintended effects of trial interventions or trial conduct	Pages 5-6
Auditing	23	Frequency and procedures for auditing trial conduct, if any, and whether the process will be independent from investigators and the sponsor	Page 8
**Ethics and dissemination**			
Research ethics approval	24	Plans for seeking research ethics committee/institutional review board (REC/IRB) approval	Page 9
Protocol amendments	25	Plans for communicating important protocol modifications (eg, changes to eligibility criteria, outcomes, analyses) to relevant parties (eg, investigators, REC/IRBs, trial participants, trial registries, journals, regulators)	Page 9
Consent or assent	26a	Who will obtain informed consent or assent from potential trial participants or authorised surrogates, and how (see Item 32)	Page 9
	26b	Additional consent provisions for collection and use of participant data and biological specimens in ancillary studies, if applicable	N/A
Confidentiality	27	How personal information about potential and enrolled participants will be collected, shared, and maintained in order to protect confidentiality before, during, and after the trial	Page 9

For peer review only - http://bmjopen.bmj.com/site/about/guidelines.xhtml

Declaration of interests	28	Financial and other competing interests for principal investigators for the overall trial and each study site	Page 11
Access to data	29	Statement of who will have access to the final trial dataset, and disclosure of contractual agreements that limit such access for investigators	Pages 9-10
Ancillary and post-trial care	30	Provisions, if any, for ancillary and post-trial care, and for compensation to those who suffer harm from trial participation	Page 10
Dissemination policy	31a	Plans for investigators and sponsor to communicate trial results to participants, healthcare professionals, the public, and other relevant groups (eg, via publication, reporting in results databases, or other data sharing arrangements), including any publication restrictions	Page 10
	31b	Authorship eligibility guidelines and any intended use of professional writers	N/A
	31c	Plans, if any, for granting public access to the full protocol, participant-level dataset, and statistical code	Page 10
**Appendices**			
Informed consent materials	32	Model consent form and other related documentation given to participants and authorised surrogates	Appendix 2
Biological specimens	33	Plans for collection, laboratory evaluation, and storage of biological specimens for genetic or molecular analysis in the current trial and for future use in ancillary studies, if applicable	N/A

*It is strongly recommended that this checklist be read in conjunction with the SPIRIT 2013 Explanation & Elaboration for important clarification on the items. Amendments to the protocol should be tracked and dated. The SPIRIT checklist is copyrighted by the SPIRIT Group under the Creative Commons "Attribution-NonCommercial-NoDerivs 3.0 Unported" license.

For peer review only

BMJ Open

TEST (Trial of Eczema allergy Screening Tests): protocol for feasibility randomised controlled trial of allergy tests in children with eczema, including economic scoping and nested qualitative study

Journal:	*BMJ Open*
Manuscript ID	bmjopen-2018-028428.R1
Article Type:	Protocol
Date Submitted by the Author:	14-Feb-2019
Complete List of Authors:	Ridd, Matthew; University of Bristol, Social & Community Medicine; University of Bristol<br>Edwards, Louisa; Simon Fraser University, Faculty of Health Sciences; Centre for Clinical Epidemiology and Evaluation (C2E2),<br>Santer, Miriam; University of Southampton, Primary Care & Population Sciences<br>Chalmers, Joanne; University of Nottingham, Dermatology<br>Waddell, Lisa; Nottingham CityCare<br>Marriage, Deborah; Bristol Royal Hospital for Children<br>Muller, Ingrid; University of Southampton, Department of Psychology<br>Roberts, Kirsty; University of Bristol School of Social and Community Medicine<br>Garfield, Kirsty; University of Bristol, Bristol Randomised Trials Collaboration<br>Coast, Joanna; Bristol University, Population Health Sciences<br>Selman, Lucy; University of Bristol School of Social and Community Medicine<br>Clement, Clare; University of Bristol, School of Social and Community Medicine; University of Bristol<br>Shaw, Ali; University of Bristol, School of Social and Community Medicine<br>Angier, Elizabeth; University of Southampton<br>Blair, Peter; University of Bristol, Centre for Academic Child Health<br>Turner, Nicholas; University of Bristol,<br>Taylor, Jodi; University of Bristol, School of Clinical Sciences<br>Kai, Joe; University of Nottingham, Division of Primary Care<br>Boyle, R; Imperial College London
**Primary Subject Heading**:	Immunology (including allergy)
Secondary Subject Heading:	Dermatology, General practice / Family practice
Keywords:	Eczema < DERMATOLOGY, Allergy < THORACIC MEDICINE, Clinical trials < THERAPEUTICS

For peer review only - http://bmjopen.bmj.com/site/about/guidelines.xhtml

Title page

Title

TEST (Trial of Eczema allergy Screening Tests): protocol for feasibility randomised controlled trial of allergy tests in children with eczema, including economic scoping and nested qualitative study

Authors

Matthew J Ridd,[1] Louisa Edwards,[2] Miriam Santer,[3] Joanne R Chalmers,[4] Lisa Waddell,[5] Deb Marriage,[6] Ingrid Muller,[2] Kirsty Roberts,[1] Kirsty Garfield,[7] Joanna Coast,[8] Lucy Selman,[7] Clare Clement,[7] Alison R G Shaw,[1] Elizabeth Angier,[3] Peter S Blair,[9] Nicholas L Turner,[7] Jodi Taylor,[7] Joe Kai,[10] Robert J Boyle[11]

[1] Population Health Sciences, University of Bristol

[2] Faculty of Health Sciences, Simon Fraser University, Burnaby, Canada

[3] Primary Care and Population Sciences, University of Southampton, Southampton, UK

[4] Centre of Evidence Based Dermatology, University of Nottingham, Nottingham, UK

[5] Nottingham CityCare Partnership, Nottingham, UK

[6] Bristol Royal Hospital for Children, University Hospitals Bristol NHS Foundation Trust, Bristol, UK

[7] Bristol Randomised Trials Collaboration, Bristol Trials Centre, University of Bristol, Bristol, UK.

[8] Health Economics at Bristol, Population Health Sciences, University of Bristol, UK

[9] Centre for Academic Child Health, Population Health Sciences, University of Bristol, UK

[10] Division of Primary Care, University of Nottingham, UK

[11] Section of Paediatrics, Imperial College London, London UK; Centre of Evidence Based Dermatology, University of Nottingham, Nottingham, UK

Corresponding author

Matthew Ridd, GP and Reader in Primary Healthcare

Postal address: Centre for Academic Primary Care, Population Health Sciences, Bristol Medical School, University of Bristol, Canynge Hall, 39 Whatley Road, Bristol BS8 2PS

Email: m.ridd@bristol.ac.uk

Telephone: 0117 331 4557

Word count

excluding title page, abstract, references, figures and tables: 3984/4000

Keywords

Atopic eczema/dermatitis, food allergy, feasibility RCT

For peer review only - http://bmjopen.bmj.com/site/about/guidelines.xhtml

ABSTRACT

Background

Early-onset eczema is associated with food allergy, and allergic reactions to foods can cause acute exacerbations of eczema. Parents often pursue dietary restrictions as a way of managing eczema and seek allergy testing for their children to guide dietary management. However, it is unclear whether test-guided dietary management improves eczema symptoms, and whether the practice causes harm through reduced use of conventional eczema treatment or unnecessary dietary restrictions. The aim of the TEST (Trial of Eczema allergy Screening Tests) study is to determine the feasibility of conducting a trial comparing food allergy testing and dietary advice versus usual care, for the management of eczema in children.

Methods and analysis

Design: a single centre, two-group, individually randomised, feasibility RCT with economic scoping and a nested qualitative study. Setting: GP surgeries in the West of England. Participants: children aged over 3 months and less than 5 years with mild to severe eczema. Interventions: allergy testing (structured allergy history and skin prick tests) or usual care. Sample size and outcome measures: we aim to recruit 80 participants and follow them up using 4-weekly questionnaires for 24 weeks. Nested qualitative study: We will conduct ~20 interviews with parents of participating children, 5-8 interviews with parents who decline or withdraw from the trial and ~10 interviews with participating GPs. Economic scoping: We will gather data on key costs and outcomes to assess the feasibility of carrying out a cost-effectiveness analysis in a future definitive trial.

Ethics and dissemination

The study has been reviewed by the Health Research Authority and given a favourable opinion by the NHS REC (West Midlands – South Birmingham Research Ethics Committee, Reference Number 18/WM/0124). Findings will be submitted for presentation at conferences and written up for publication in peer-reviewed journals.

Trial registration

ISRCTN: 15397185 (30 July 2018)

Word count: 296/300

Strength and limitations

- This is the first RCT exploring test-guided dietary management for treating eczema to be done in a primary care setting, where most children with eczema are diagnosed and managed in the UK.
- Data on the processes and outcomes that are being collected will help determine the feasibility of a definitive trial and associated economic evaluation.
- The nested qualitative study will help to interpret and explain the quantitative feasibility findings and to generate new knowledge around the issues of food allergy, allergy tests and dietary modification in children with eczema, from the perspective of parents and GPs

For peer review only - http://bmjopen.bmj.com/site/about/guidelines.xhtml

- The study is being conducted in a single centre in the West of England, which may limit the generalisability of the findings.

For peer review only

For peer review only - http://bmjopen.bmj.com/site/about/guidelines.xhtml

Main text

INTRODUCTION

Background and rationale

Childhood eczema is a common long-term condition characterised by dry and itchy skin. In accordance with the recommended nomenclature of the World Allergy Organisation, we use the label “eczema” to refer to the clinical phenotype of atopic eczema/dermatitis.[1]

Eczema affects around 20% of pre-school age children; 60% of these develop symptoms in the first year of life and 90% by five years of age.[2] In the UK. most children with eczema are diagnosed and managed in primary care with a combination of emollients and topical corticosteroids. Having eczema can significantly impact the quality of life of the affected child and their family. Treatment adherence can be problematic for numerous reasons, including parents/carers (hereafter, “parents”) seeking a “cure” through dietary exclusions for possible food allergy rather than “control” through long-term use of topical treatments.[3-5]

Eczema is associated with food allergy, especially early-onset, troublesome eczema,[6] and parents of children with eczema often try dietary exclusions in an attempt to reduce symptom severity and may seek allergy testing to guide such dietary exclusions. Allergic reactions to food can cause an acute exacerbation of eczema, either as part of an IgE-mediated reaction or as an isolated non-IgE mediated reaction to a food (see Table 1). Parents’ suspicions of food allergies in general and especially with respect to eczema have low specificity. Depending on the specific population studied and the definitions used, 15-36% of children with eczema compared to about 6% of the general population have a food sensitivity (a ‘positive’ test result, without clinical symptoms) or allergy.[7] Clinical practice in offering allergy tests to parents of children with eczema varies significantly, with many allergy clinics routinely “screening” for associated food allergies, but few primary care services offering testing in the absence of a history suggesting an IgE-mediated reaction to a food.

A Cochrane review[8] of dietary exclusions for adults and children with eczema published in 2008 did not find any evidence of benefit for exclusion diets in unselected populations (i.e. those without clinically suspected food allergies), but did identify one trial which suggested that infants with suspected egg allergy who have positive specific IgE to eggs may benefit from an egg-free diet.[9] While this suggests that test-guided dietary management may be worthwhile, both this and two other subsequently published systematic reviews[10 11] have called for better-designed and conducted trials. We have not identified any economic evaluations in this area and while concerns about food allergy have been raised during in-depth interviews of parents’ general experiences of looking after children with eczema,[3-5] and have arisen as an important concern for parents in online discussion forums,[12] we are not aware of any qualitative work specifically exploring this issue.

Aim and objectives

The aim of the study to determine the feasibility of conducting a trial comparing test-guided dietary management versus usual care, for the management of eczema in children.

The objectives are to explore the following factors that will determine the feasibility and inform the design of a future, full-scale clinical and cost-effectiveness RCT:

- participant recruitment (including numbers potentially eligible), retention and adherence to allocation/dietary advice;

For peer review only - http://bmjopen.bmj.com/site/about/guidelines.xhtml

- outcome completion rates; and
- logistics of of trial processes and their acceptability to participants

Trial design

TEST is a single centre, two-group, individually randomised, feasibility RCT[13] with economic scoping and nested qualitative study.

METHODS AND ANALYSIS

Study setting

Primary care (GP surgeries) in the West of England.

Recruitment

The stages of participant recruitment are shown in Figure 1.

We will identify children aged between 3 months and 5 years with eczema via an electronic query-based records search developed by the research team and run by practice staff at the GP surgeries. A GP or a delegated member of the practice team will screen the search results for inclusion/exclusion criteria and any other known adverse medical or social circumstance that would make invitation to the study inappropriate. Surgeries will be asked to provide the research team with the number of participants excluded, along with a brief reason for exclusion. Parents of potentially eligible children will be sent an invitation pack, comprising an invitation letter, study flyer and response to invitation to participate form. In addition, we will also recruit participants opportunistically, by placing posters in participating GP surgeries and supplying study flyers for practice staff and health visitors to hand out.

Interested families will be asked to complete a brief screening questionnaire that the research team will use to assess initial eligibility. Parents of potentially eligible participants will be contacted by a member of the research team to explain more about the study and schedule a baseline assessment at a participating GP surgery. At this visit, consent will be received, baseline data collected, and randomisation undertaken.

Eligibility and allocation

Inclusion and exclusion criteria are summarised in Table 2.

Individual randomisation to intervention or comparator groups (1:1 ratio), stratified by age (less than 1 year, 1 year to less than 2 years, 2 years and above) and eczema severity (mild, moderate/severe)[14] and blocked within strata, using the Bristol Randomised Trials Collaboration (BRTC) web-based system. Allocation concealment will be ensured, as the CSO will not randomise the participant until all baseline measurements have been completed.

Interventions

All participants allocated to the intervention group will undergo a structured allergy history, skin prick tests and will be given dietary advice. Where the child's history and the results of the skin prick test results are equivocal, participants will be offered repeat skin prick tests and/or oral food challenges and/or home dietary trial of exclusion or inclusion. Repeat skin prick tests will be done either at the same appointment or 12 weeks after the baseline appointment. Advice will be tailored accordingly for mothers who are breastfeeding and/or babies who have not yet been weaned.

For peer review only - http://bmjopen.bmj.com/site/about/guidelines.xhtml

- **Structured allergy history:** The researcher (Clinical Studies Officer, CSO) will first take a structured allergy history. There are recommendations for what a structured allergy history should comprise,[15] but no validated questionnaires. With reference to published guidance,[7 16] we have therefore modified questionnaires developed for the BEEP trial.[17] These questions capture relevant symptoms (skin, respiratory and gastrointestinal) and timing of onset in relation to ingestion of the study foods.
- **Skin prick tests:** The CSO will carry out the skin prick tests using commercial extracts of cow's milk, hen's egg (white), wheat, peanut, cashew and codfish, along with positive (1.0% histamine) and negative (0.9% saline) controls.[18 19] 1 mm shouldered sterile lancets will be used (ALK, Denmark) and the diameter (mean of longest and shortest perpendicular axis if ovoid or irregular) of any wheal reaction, resulting from the release of histamine and other mediators, will be measured after 15 minutes.[20]
- **Oral food challenge:** Supervised open food challenges will be undertaken at Bristol Royal Children's Hospital, using a modified PRACTALL dosing schedule and criteria for interpretation of challenge outcome,[21] usually within 1-2 weeks of the baseline appointment. Consent specifically for oral food challenge will be received and standard hospital protocols for each allergen will be followed. For pragmatic and cost reasons, they will be unblinded as in normal clinical practice, rather than the diagnostic "gold standard" of the double-blind, placebo-controlled food challenge.[22]
- **Home dietary trial:** For participants whose history and investigation findings suggest the possibility of a delayed-type reaction, they will be advised to either exclude or reintroduce (as appropriate to their path in the study) the possible allergen from/into their diet over a 2-4 week period, as per current clinical practice.[16]
- **Dietary advice:** An algorithm describing the approach to the interpretation of the structured allergy history, skin prick test results, +/- oral food challenge, and consequent dietary guidance, will be developed and tested as part of this feasibility study, guided in part by published guidance on diagnosis of food allergy in epidemiological studies.[23] All participants' results will also be reviewed by an expert allergy panel and dietary advice relayed to their family accordingly.

Participants in the comparator group will receive care as usual, as described in the NICE eczema and allergy in children guidelines and will not receive any additional assessments or tests.[16 24] Any allergy tests and subsequent advice will be monitored as part of this feasibility study.

Regardless of allocation, all care after randomisation, including investigations and/or referrals for possible food allergies, will remain with the participant's GP.

Outcomes

The primary outcome is the feasibility of conducting the trial (recruitment, retention, contamination) and collecting the required data (appendix 1). A complete schedule of data collection can be found in Table 3. The feasibility of collecting data in the key domains that are likely to be used in the definitive trial (symptoms, clinical signs, long-term control and quality of life, as recommended by the core outcome group for eczema, HOME)[25] will be assessed:

- Patient Orientated Eczema Measure[26] (POEM, proposed primary outcome in the definitive trial) completed by proxy (parent report) captures symptoms of importance to parents and patients.[27] Emerging data suggests that monthly, as opposed to weekly, collection is adequate for the purpose of capturing long-term control.[28] It demonstrates good validity, repeatability and responsiveness to change.[29 30]

For peer review only - http://bmjopen.bmj.com/site/about/guidelines.xhtml

- Eczema Area Severity Index (EASI),[31] a validated scoring system that grades the physical signs of eczema. Administered by a trained researcher, it will provide an independent assessment of eczema severity.
- Long-term control will be captured by repeated, four weekly, administration of POEM.
- Disease-specific (Atopic Dermatitis Quality of Life, ADQoL;[32] Infant Dermatitis Quality of Life, IDQoL[33 34]) and generic (Children's Health Utility 9D, CHU-9D[35 36]) quality of life measures will be collected at baseline, weeks 8 and 24. The CHU-9D is currently validated for children aged 7 years and over,[37] so additional guidance notes and validation questions are included.

With consent, participants' electronic medical records (EMR) will be reviewed at 24 weeks (from four weeks before and for the duration of time in the study) for data on NHS consultations, treatments, referrals for eczema/allergies and relevant prescribed medications.

For participants in the intervention group, the following data will also be collected:

- Structured allergy history
- Results of Skin Prick Test (SPT) +/- Oral Food Challenge (OFC) +/- home dietary trial

Data collection methods and retention

Baseline data will be collected by the CSO using paper case report forms (CRFs). Parents will be given the option of completing follow-up questionnaires either online or on paper. In recognition of participant's time and to encourage retention in the study/data collection, parents of participants will be offered £10 vouchers at the baseline and around the 24-week visit. We will also offer the child a small gift of about £5 in value.

Blinding

It is not possible to blind participants, their families or treating clinicians to allocation. The research team will notify the appropriate GP surgery of the participant's allocation and the outcome of any tests/investigations and food allergy diagnoses.

The CSO undertaking the baseline visit cannot be blinded, but all baseline data (including EASI) will be collected before randomisation. If possible, the follow-up visit will be done by a different CSO, who will be blinded to allocation. Parents will be asked not to disclose allocation to the CSO doing the follow-up visit. CSO blinding will be monitored by means of self-report.

Participant timeline

Participants are in the study for 24 weeks, from the baseline until the follow-up visit. Figure 1 provides an overview of participants' pathway through the study.

Sample size

As this is a feasibility RCT, a formal sample size calculation is not appropriate. On a pragmatic basis, we have determined that 80 children (approximately 40 in each group) will be sufficient to provide estimates of recruitment, retention, adherence and assessment of contamination within GP surgeries and between groups. This is broadly in-line with published "rules of thumb".[38 39]

For peer review only - http://bmjopen.bmj.com/site/about/guidelines.xhtml

Data management

Data will be entered onto the study database. The system will incorporate data entry and validation rules to reduce data entry errors, and management functions to facilitate auditing and data quality assurance.

Statistical methods

The aim will be to determine the feasibility of undertaking the main trial and explore acceptability. We will report our findings following the pilot and feasibility extension of the CONSORT guidance (2010), including a CONSORT diagram, descriptive and summary statistics, along with all important harms or unintended effects in each group.

Descriptive statistics will be used to compare recruitment, retention, adherence and contamination rates overall and between the two groups; and in the intervention group, test results and adherence to dietary advice. Completion rates, average score and distributions (as appropriate) will be reported for the proposed outcomes in the main trial, e.g. POEM and EASI.

Economic scoping

We will gather data on key costs and outcomes to assess the feasibility of carrying out a cost-effectiveness analysis from the primary perspective of the NHS and from a wider perspective including parental costs and time off work.

Data on healthcare contacts and prescribed medications will be extracted from EMRs. Additional healthcare contacts, information about parental out-of-pocket expenses and time off work will be collected using four-weekly parent-completed questionnaires. The overall level of missing data will be recorded and the pattern of missing data, by item, will be explored. Relevant unit costs will be identified and, once resource-use has been costed, we will identify which items are important cost drivers. The resources required for the intervention will be identified and the feasibility of costing these established.

NICE recommends the use of Quality-adjusted life-years (QALYs) as the preferred outcome measure in economic evaluations, but it is unclear what the most appropriate underlying measure is for this population in estimating QALYs. Therefore, we will test feasibility and validity of using both condition-specific (ADQoL)[32] and generic (CHU-9D) [35 36] preference-based health-related quality of life measures in children (measured at baseline, eight and 24 weeks) to estimate QALYs. The CHU-9D is currently validated for children aged 6 and over, with pilot versions for those aged 5-7 and additional guidance notes and validation questions for those under 5. One key component of the economic work will be to determine the feasibility of using the CHU-9D in this pre-school age group.

Nested qualitative study

The aims of the qualitative study are to help interpret and explain the quantitative feasibility findings (including experience and acceptability of study processes/intervention); and to generate new knowledge around the issues of food allergy, allergy tests and dietary modification in children with eczema, from the perspective of parents and GPs.

GPs at participating surgeries will be asked to complete a brief questionnaire and all parents and GPs will be asked whether they are willing to be contacted to take part in an interview. Semi-structured qualitative interviews will be conducted with a sample of trial parents and GPs from participating

For peer review only - http://bmjopen.bmj.com/site/about/guidelines.xhtml

surgeries, using topic guides developed based on study aims and input from the Trial Management Group (TMG).

Parents will be selected purposively to ensure diversity in relevant characteristics: trial group (intervention or comparator) with oversampling of the intervention group; eczema severity according to POEM (mild/moderate (<17) vs severe (≥17)); socio-economic status (assessed via postcode, using the Index of Multiple Deprivation Decile (categories: high (8-10)/medium (5-7)/low (1-4));[40] for mothers, whether currently breastfeeding; and length of time in the trial (shortly after baseline visit or OFC, or later in the trial). GPs will be purposively sampled to capture variation in GP surgery deprivation decile,[40] length of time in the trial, number of years' experience as a GP and confidence in managing children with eczema (assessed via a single item scored 1 (low) – 10 (high)). Sampling will stop when we have sufficient information power relevant to the study aims;[41] we anticipate a total of 20 parent and 10 GP interviews.

In addition, we will conduct brief telephone interviews with 5-8 parents who are ineligible to participate, decline to take part, or withdraw during the trial but indicate that they are willing to discuss reasons why. This information may provide valuable data to inform the design of a future definitive trial.

Interviews will be conducted by an experienced qualitative researcher, either by telephone or face-to-face, depending on the preference of the interviewee, audio-recorded (with permission) and transcribed verbatim. All interviewees will receive an information sheet and consent form to read in advance of the interview. Written informed consent will be taken prior to face-to-face interviews, and verbal consent will be taken for telephone interviews.

Data analysis of interview transcripts will take place alongside data collection and inform further data collection. We will conduct a thematic analysis, using both inductive and deductive coding (informed by the Common Sense model).[42]

Monitoring, safety and audit

Because this is a low-risk feasibility trial, the trial is over seen by a joint Trial Steering/Data Monitoring Committee (TS/DM-C) which is comprised of four independent members: a chairperson, a biostatistician, a clinician, and a patient representative (parent of child with eczema). Their role will be to provide overall supervision of the trial on behalf of the funder, with a focus on progress of the trial, adherence to the protocol, patient safety and consideration of new information.

Adverse events will be collected in the CRFs and by parent/clinician report and reported to the TMG and TS/DM-C. Possible serious adverse events include:

- severe localised reaction (redness, swelling, itch) to one or more SPTs necessitating medication and/or hospitalisation; and/or
- anaphylactic reaction (generalised flushing of the skin, hives, swelling of throat and mouth, difficulty in swallowing or speaking, tachycardia, severe asthma, abdominal pain and/or nausea and vomiting, hypotension and/or collapse and unconsciousness) requiring medication +/- hospitalisation (SPTs or OFC).

The sponsor organisation is the University of Bristol.

For peer review only - http://bmjopen.bmj.com/site/about/guidelines.xhtml

PRE-GRANT APPLICATION SURVEY

An online survey of parents of children with eczema informed the study design. It was promoted via social media and partner eczema and allergy websites between 10-27 October 2016. We received 152 responses, 97% (145/150) female with a mean age of 38.8 years. The median number of children with eczema was 1 (interquartile range 1,2) and the mean POEM score (for the worst affected child, where more than one child with eczema) was 11.7 (SD 7.6). 74% (108/146) had one or more food allergies, the most common being peanut, egg and cow's milk. 71.3% (77/108) had received allergy tests and been given advice by a healthcare professional and 17.6% (19/108) based their report on their observation of symptoms/reaction alone.

Participants were asked "In a study that compares the effect of doing allergy tests or giving advice on avoiding certain foods in children, what would be the single most important thing that this kind of study could tell you about?" Overall, 37% (56/151) chose "Reduce the risk of a sudden or severe allergic reaction". However, among those children without a reported food allergy (the group of interest in this study), 44% (16/36) chose "Reduce day-to-day severity of eczema". Consequently, we included eczema severity as a key clinical outcome.

Regarding the then proposed study, 96% (144/150) said they would be willing for their child to have an allergy test, with 67.1% (100/149) identifying skin prick as their "first choice" option for testing for allergy, and 54.3% (82/151) saying a blood test was an acceptable "second choice". Other participants said they would refuse (4.0% skin prick, 8.5% blood test) or did not know (2.0% skin prick, 2.6% blood test). Further information about the limitations of both types of test (risk of false reassurance or worry) did not change the opinion of the majority (72.5%, 108/149) of respondents. 56.9% (74/130) said that based on the clinical history and allergy test, they would be willing to avoid that food for at least 24 months. These findings provided reassurance as to the acceptability of the intervention, which includes skin prick tests and the possibility of having to exclude foods for at least several months.

PUBLIC AND PATIENT INVOLVEMENT

The James Lind Alliance eczema research priority setting partnership (2013) identified the following questions: "What role might food allergy tests play in treating eczema?" and "What is the role of [exclusion] diets in treating eczema?",[43] which a follow-on definitive trial could begin to address.

Two mothers of children with eczema (Gray & McMeechan) are members of the TMG and regularly attend the meetings. They have commented on the research proposal and study paperwork, and their suggestions around nomenclature and reducing data burden on participants have been incorporated. A lay member also sits on TS/DM-C.

We have established and met with a wider PPI advisory group. It first met towards the beginning of the research to discuss data burden and the design of patient facing materials. At a subsequent meeting, study progress and challenges were discussed. One more meeting is planned towards the end of the study, to inform write-up and dissemination of findings.

For peer review only - http://bmjopen.bmj.com/site/about/guidelines.xhtml

ETHICS AND DISSEMINATION

Research ethics approval

The study has been reviewed by the Health Research Authority and given a favourable opinion by the NHS REC (West Midlands – South Birmingham Research Ethics Committee, Reference Number 18/WM/0124).

Protocol amendments

Any amendments to the Protocol will be reported accordingly to the regulatory bodies, with a full copy of the current protocol available for download from the study website. Amendments to date are listed in appendix 1.

Consent or assent

Written consent for taking part in the trial will be received by a CSO from the parent or guardian of the participant at their baseline appointment, which takes place in a participating GP practice. Consent is also sought to contact participants regarding possible interview in the nested qualitative study; and for the re-use of the anonymised data in future research for purposes not related to this study, including as publicly available "open data" (see Appendix 2). Consent for oral food challenges is received by the hospital nurse undertaking the procedure.

Confidentiality and access to data

The database and randomisation system will protect patient information in line with the data protection legislation. Trial staff will ensure that participants' anonymity is maintained through protective and secure handling and storage of patient information at the trial centre.

The Chief Investigator (CI) will have access to and act as custodian of the full dataset, which will be made available to the TS/DM-C if requested to verify the validity of the findings.

Ancillary and post-trial care

Participants requiring follow-up beyond their six months in the study will be referred by their GP to their local allergy clinic.

Dissemination and data sharing

Study progress, outputs and a summary of findings will be made available via a study website and Twitter account; and summaries distributed to participating families and GP surgeries. Findings will be submitted for presentation at conferences and written up for publication in a peer-reviewed journal(s), which may include mixed-method triangulation and integration of the quantitative and qualitative findings.

No later than three years after the completion of the study, we will make available a completely deidentified data set to an appropriate data archive for sharing purposes.

DISCUSSION

There are wide variations in provision of allergy testing for children with eczema. Parental concern and clinician uncertainty about the role of food allergy in eczema has been highlighted as a barrier to effective treatment.[44] Up to 70% of parents make significant modifications to their child's diet, often

For peer review only - http://bmjopen.bmj.com/site/about/guidelines.xhtml

without professional advice,[45] even if the child has only mild eczema. Many parents turn to the internet for advice,[12 46] or purchase self-test allergy kits which are not validated and not recommended.[16]

It is uncommon for allergy tests to be undertaken in primary care but in principle, allergy testing (in the form of skin prick tests) and advice could be routinely delivered in primary care, but evidence is required to demonstrate both the feasibility and value of doing so. An RCT is needed to determine the clinical and cost-effectiveness of food allergy testing and advice in primary care, on severity of eczema in children. There are potentially significant benefits for the NHS of improving long-term eczema management, avoiding serious allergic reactions, and targeting child nutrition. This study will provide important data to first, determine the feasibility of a large, definitive trial; and second, to inform its design.

The full/most up-to-date version of the protocol is available to download from the study website. The first participant was randomised in September 2018 and recruitment is on-going. Follow-up is expected to be complete by September 2019. We expect to report in early 2020.

REFERENCES

1. Johansson S, Bieber T, Dahl R, et al. Revised nomenclature for allergy for global use: Report of the Nomenclature Review Committee of the World Allergy Organization, October 2003. *J Allergy Clin Immunol* 2004;113:832-36.
2. Eichenfield LF, Tom WL, Chamlin SL, et al. Guidelines of care for the management of atopic dermatitis: Part 1. Diagnosis and assessment of atopic dermatitis. *Journal of the American Academy of Dermatology* 2014;70(2):338-51. doi: http://dx.doi.org/10.1016/j.jaad.2013.10.010
3. Santer M, Burgess H, Yardley L, et al. Experiences of carers managing childhood eczema and their views on its treatment: a qualitative study. *British Journal of General Practice* 2012;62(597):e261-e67. doi: 10.3399/bjgp12X636083
4. Santer M, Burgess H, Yardley L, et al. Managing childhood eczema: qualitative study exploring carers' experiences of barriers and facilitators to treatment adherence. *Journal of Advanced Nursing* 2013;69(11):2493-501. doi: 10.1111/jan.12133
5. Powell K, Le Roux E, Banks J, et al. GP and parent dissonance about the assessment and treatment of childhood eczema in primary care: a qualitative study. *BMJ Open* 2018;8(2) doi: 10.1136/bmjopen-2017-019633
6. Martin PE, Eckert JK, Koplin JJ, et al. Which infants with eczema are at risk of food allergy? Results from a population-based cohort. *Clinical & Experimental Allergy* 2015;45(1):255-64. doi: 10.1111/cea.12406
7. Cox H, Lloyd K, Williams H, et al. Emollients, education and quality of life: the RCPCH care pathway for children with eczema. *Archives of Disease in Childhood* 2011;96(Suppl 2):i19-i24.
8. Bath-Hextall F, Delamere FM, Williams HC. Dietary exclusions for improving established atopic eczema in adults and children: systematic review. *Allergy* 2009;64(2):258-64.
9. Lever R, MacDonald C, Waugh P, et al. Randomised controlled trial of advice on an egg exclusion diet in young children with atopic eczema and sensitivity to eggs. *Pediatric Allergy and Immunology* 1998;9(1):13-19. doi: 10.1111/j.1399-3038.1998.tb00294.x
10. Chafen J, Newberry SJ, Riedl MA, et al. Diagnosing and managing common food allergies: A systematic review. *JAMA* 2010;303(18):1848-56. doi: 10.1001/jama.2010.582
11. Nankervis H, Thomas K, Delamere F, et al. Scoping systematic review of treatments for eczema. *Programme Grants Appl Res* 2016;4(7)

For peer review only - http://bmjopen.bmj.com/site/about/guidelines.xhtml

12. Halls A, Nunes D, Muller I, et al. 'Hope you find your 'eureka' moment soon': a qualitative study of parents/carers' online discussions around allergy, allergy tests and eczema. *BMJ Open* 2018;8(11) doi: 10.1136/bmjopen-2018-022861
13. Eldridge SM, Lancaster GA, Campbell MJ, et al. Defining Feasibility and Pilot Studies in Preparation for Randomised Controlled Trials: Development of a Conceptual Framework. *PLoS ONE* 2016;11(3):e0150205. doi: 10.1371/journal.pone.0150205
14. Charman C, Venn A, Ravenscroft J, et al. Translating Patient-Oriented Eczema Measure (POEM) scores into clinical practice by suggesting severity strata derived using anchor-based methods. *Br J Dermatol* 2013;169(6):1326-32. doi: 10.1111/bjd.12590
15. RCPCH Allergy Care Pathways Project. Taking an allergy focused clinical history. https://www.rcpch.ac.uk/sites/default/files/Taking_an_Allergy_Focused_Clinical_History_-_Allergy_Care_Pathways_Project.pdf [Accessed 07.12.18]
16. NICE. Food allergy in children and young people - Diagnosis and assessment of food allergy in children and young people in primary care and community settings. London: National Institute for Health and Clinical Excellence, 2011.
17. Chalmers JR, Haines RH, Mitchell EJ, et al. Effectiveness and cost effectiveness of daily all over body application of emollient during the first year of life for preventing atopic eczema in high risk children (The BEEP trial): study protocol for a randomised controlled trial. Trials 18: 343 DOI : 10.1186/s13063-017-2031-3
18. Burks AW, James JM, Hiegel A, et al. Atopic dermatitis and food hypersensitivity reactions. *The Journal of Pediatrics* 1998;132(1):132-36. doi: http://dx.doi.org/10.1016/S0022-3476(98)70498-6
19. Niggemann, Sielaff, Beyer, et al. Outcome of double-blind, placebo-controlled food challenge tests in 107 children with atopic dermatitis. *Clinical & Experimental Allergy* 1999;29(1):91-96. doi: 10.1046/j.1365-2222.1999.00454.x
20. Heinzerling L, Mari A, Bergmann K-C, et al. The skin prick test – European standards. *Clinical and Translational Allergy* 2013;3(1):3. doi: 10.1186/2045-7022-3-3
21. Sampson HA, van Wijk RG, Bindslev-Jensen C, et al. Standardizing double-blind, placebo-controlled oral foodchallenges: American Academy of Allergy, Asthma & Immunology–European Academy of Allergy and Clinical Immunology PRACTALL consensus report. *J Allergy Clin Immunol* 2012;130 doi: 10.1016/j.jaci.2012.10.017
22. Ricci G, Dondi A, Patrizi A. Useful tools for the management of atopic dermatitis. *Am J Clin Dermatol* 2009;10:287-300.
23. Grabenhenrich LB, Reich A, Bellach J, et al. A new framework for the documentation and interpretation of oral food challenges in population-based and clinical research. *Allergy* 2017;72(3):453-61. doi: 10.1111/all.13049
24. National Institute for Health and Clinical Excellence. CG057: Atopic eczema in children - management of atopic eczema in children from birth up to the age of 12 years. London: RCOG Press, 2007.
25. Schmitt J, Spuls P, Boers M, et al. Towards global consensus on outcome measures for atopic eczema research: results of the HOME II meeting. *Allergy* 2012;67:1111-17. doi: 10.1111/j.1398-9995.2012.02874.x
26. Chalmers JR, Simpson E, Apfelbacher CJ, et al. Report from the fourth international consensus meeting to harmonize core outcome measures for atopic eczema/dermatitis clinical trials (HOME initiative). *BJD* 2016 Jul; 175(1): 69-79 doi: 10.1111/bjd.14773
27. Charman CR, Venn AJ, Williams HC. The Patient-Oriented Eczema Measure: Development and Initial Validation of a New Tool for Measuring Atopic Eczema Severity From the Patients' Perspective. *Archives of Dermatology* 2004;140(12):1513-19.
28. Thomas K, Bradshaw L, Montgommery A, et al. Using existing trial data to inform the development of core outcome sets and improve efficiencies in research. *Trials* 2015;16(Suppl 2):O74.

For peer review only - http://bmjopen.bmj.com/site/about/guidelines.xhtml

29. Schmitt J, Langan S, Williams HC. What are the best outcome measurements for atopic eczema? A systematic review. *Journal of Allergy and Clinical Immunology* 2007;120(6):1389-98. doi: 10.1016/j.jaci.2007.08.011
30. Gaunt DM, Metcalfe C, Ridd M. The Patient-Oriented Eczema Measure in young children: responsiveness and minimal clinically important difference *Allergy* 2016; 71: 1620–1625 doi: 10.1111/all.12942
31. Hanifin JM, Thurston M, Omoto M, et al. The eczema area and severity index (EASI): assessment of reliability in atopic dermatitis. *Experimental Dermatology* 2001;10(1):11-18.
32. Stevens KJ, Brazier JE, McKenna SP, et al. The development of a preference-based measure of health in children with atopic dermatitis. *BJD* 2005;153(2):372-77.
33. Basra MKA, Gada V, Ungaro S, et al. Infants' Dermatitis Quality of Life Index: a decade of experience of validation and clinical application. *BJD* 2013;169(4):760-68. doi: 10.1111/bjd.12563
34. Lewis-Jones MS, Finlay AY, Dykes PJ. The Infants Dermatitis Quality of Life Index. *BJD* 2001;144(1):104-10.
35. Stevens K. Assessing the performance of a new generic measure of health-related quality of life for children and refining it for use in health state valuation. *Applied Health Economics and Health Policy* 2011;9(3):157-69.
36. Stevens K, Katherine S. Valuation of the Child Health Utility 9D Index. *Pharmacoeconomics* 2012;30:729-47.
37. Canaway AG, Frew EJJ. Measuring preference-based quality of life in children aged 6–7 years: a comparison of the performance of the CHU-9D and EQ-5D-Y—the WAVES Pilot Study. *Quality of Life Research* 2013;22(1):173-83. doi: 10.1007/s11136-012-0119-5
38. Browne R. On the use of a pilot sample for sample-size determination. *Stat Med* 1995;14(17):1933 - 40.
39. Teare M, Dimairo M, Shephard N, et al. Sample size requirements to estimate key design parameters from external pilot randomised controlled trials: a simulation study. *Trials* 2014;15(1):264.
40. Smith T, Noble M, Noble S, et al. The English Indices of Deprivation 2015. London, 2015.
41. Malterud K, Siersma VD, Guassora AD. Sample Size in Qualitative Interview Studies: Guided by Information Power. *Qualitative Health Research* 2016;26(13):1753-60. doi: 10.1177/1049732315617444
42. Hagger MS, Orbell S. A Meta-Analytic Review of the Common-Sense Model of Illness Representations. *Psychology & Health* 2003;18(2):141-84. doi: 10.1080/088704403100081321
43. Batchelor JM, Ridd MJ, Clarke T, et al. The Eczema Priority Setting Partnership: a collaboration between patients, carers, clinicians and researchers to identify and prioritize important research questions for the treatment of eczema. *BJD* 2013;168(3):577-82. doi: 10.1111/bjd.12040
44. Le Roux E, Powell K, Banks JP, et al. GPs' experiences of diagnosing and managing childhood eczema: a qualitative study in primary care. *BJGP* 2018;68(667):e73-e80. doi: 10.3399/bjgp18X694529
45. Chan JP, Ridd MJ. Carer and patient beliefs and practices regarding the role of food allergy in eczema: cross-sectional survey. *Clinical & Experimental Dermatology* In Press
46. Santer M, Muller I, Yardley L, et al. 'You don't know which bits to believe': qualitative study exploring carers' experiences of seeking information on the internet about childhood eczema. *BMJ Open* 2015;5(4) doi: 10.1136/bmjopen-2014-006339
47. Werfel T, Erdmann S, Fuchs T, et al. Approach to suspected food allergy in atopic dermatitis. *JDDG: Journal der Deutschen Dermatologischen Gesellschaft* 2009;7(3):265-71. doi: 10.1111/j.1610-0387.2008.06901.x

For peer review only - http://bmjopen.bmj.com/site/about/guidelines.xhtml

For peer review only

STATEMENTS

Acknowledgements

This study was designed and is being delivered in collaboration with the Bristol Randomised Trials Collaboration (BRTC), a UKCRC registered clinical trials unit which, as part of the Bristol Trials Centre, is in receipt of National Institute for Health Research CTU support funding.

The study was developed with support from UK Dermatology Clinical Trials Network (UK DCTN). The UK DCTN is grateful to the British Association of Dermatologists and the University of Nottingham for financial support of the Network.

The TMG would like to thank the early input of Professor Hywel Williams (Centre for Evidence Based Dermatology, University of Nottingham), Dr Sandra Hollinghurst and Dr Jeremy Horwood (University of Bristol) and Dr Kate Grimshaw (University of Southampton) for their advice on study design and delivery.

We are grateful to the Centre for Evidence Based Dermatology (www.nottingham.ac.uk/research/groups/cebd), Nottingham Eczema Support Group for Carers of Children with Eczema (www.nottinghameczema.org.uk/), the National Eczema Society (www.eczema.org) and Allergy UK (www.allergyuk.org), Anaphylaxis Campaign (www.anaphylaxis.org.uk) for their helping in promoting the pre-grant PPI survey.

Trial steering and data monitoring committee members: Professor Carl Heneghan (chair), Dr David Gillespie (statistician), Dr Joanne Walsh (GP with specialist interest in allergy) and Kate Sykes (parent of child with eczema and food allergy).

Declaration of interests

MR: No financial interests; convenes the NIHR SPCR Allergy working group; and was a member of the NICE Quality Standard 44 for Atopic eczema in under 12s and RCPCH "Care pathway for children with eczema" groups.

LW: Direct – financial: write articles, attend round table infant formula company meetings and present at sponsored lectures relating to food allergy; received infant formula company sponsorship to attend national/international allergy related conferences/ meetings. Runs a private practice (Food Allergy Nottingham Service Ltd, 2013-) in addition to my NHS role (Feb 2012-). BDA cow's milk allergy course facilitator (2018-). Direct – non-financial: member of RCPCH faculty for tier 3 paediatric allergy course (2018-); member of Allergy UK health advisory board (2015-); member of iMAP implementation team (2017-); produce food allergy related dietary information for BDA food allergy group (2014-); NICE Expert adviser relating to paediatric food allergy and gastro-oesophageal reflux (2017-2020); previous member of NICE food allergy guidelines GDG and RCPCH food allergy care pathway (2010-2011)

RJB: RJB has received honoraria for participating in advisory boards for ALK-Abello who manufacture allergy diagnostics and treatments, and DBV technologies who manufacture a food allergy treatment.

For peer review only - http://bmjopen.bmj.com/site/about/guidelines.xhtml

Funding

The study is funded by National Institute for Health Research (NIHR) School for Primary Care Research (project 383). MR is funded by an NIHR Post-Doctoral Research Fellowship (PDF-2014-07-013). The views expressed in this publication are those of the authors and not necessarily those of the NHS, the National Institute for Health Research or the Department of Health and Social Care.

Contributorship statement

MR conceived the study idea in collaboration with RB, MS, JCh, IM and LE; MR, RB, MS, JCh, IM, LE, ARGS, DM, KR, KG, PB and JK developed the initial study design, with later input from JCo, LS, LW, EA and JT. Specific advice was given by PB and NT on trial design and medical statistics; AH, LS and CC on the nested qualitative study; and KG and JCo on the economic scoping. All the authors contributed to the drafting of the study protocol, led by MR, and approved the final manuscript.

Study website and social media

www.bristol.ac.uk/eczema-allergy-study and Twitter: @eczema-allergy

For peer review only

TABLES

Table 1: IgE and non-IgE mediated food allergy

- The World Allergy Organisation defines food allergy as an immune-mediated hypersensitivity reaction to food and may be divided into Immunoglobulin E (IgE) mediated and non-IgE mediated reactions.[1]
- IgE-mediated food allergy involves immediate hypersensitivity (typically within 5-30 minutes of ingestion and always within 2 hours) through the action of mast cells. It can be reliably diagnosed when there is a typical history of reaction within 1-2 hours of exposure and demonstration of specific IgE to the relevant food on blood or skin prick testing.
- Non-IgE mediated food allergy is delayed (between 2-48 hours post ingestion) and thought to be caused by an aberrant T-cell response. It is more difficult to diagnose as there are no reliable diagnostic tests other than dietary exclusions and re-introduction.[47]

Table 2: Participant eligibility criteria

Inclusion criteria are:

- child aged between 3 months and 5 years with eczema diagnosed by an appropriately qualified healthcare professional (registered doctor, nurse or health visitor)
- Patient Orientated Eczema Measure (POEM) score of >2
- consent given by a person with parental responsibility for the participant

Exclusion criteria are:

- child with medically-diagnosed food allergy, awaiting referral/investigations for possible food allergy, or had previous investigations for food allergy (not including home testing)
- the person responsible for consent has insufficient written English to complete the outcome measures, or has another child already taking part in the trial.

For peer review only

For peer review only - http://bmjopen.bmj.com/site/about/guidelines.xhtml

Table 3: Schedule of enrolment, interventions and assessments

	Study period									
	Enrolment	Allocation	Post-allocation							Close-out
		V_0	Follow-up questionnaires						V_1	
Week		0	4	8	12	16	20	24	24	
Parent-completed										
Screening questionnaire	●									
Demographics and medical history		●								
POEM	●	●	●	●	●	●	●	●		
Other eczema symptoms†		●	●	●	●	●	●	●		
Other possible symptoms of food allergy				●				●		
Diet of child (and breast-feeding mother)			●	●	●	●	●	●		
Health service utilisation			●	●	●	●	●	●		
Out-of-pocket expenses/time off work			●	●	●	●	●	●		
ADQoL		●		●				●		
CHU-9D		●		●				●		
IDQoL		●		●				●		
Parental anxiety (GAD-7)		●						●		
Exit questionnaire								●		
Researcher-administered										
UK Diagnostic criteria for atopic dermatitis		●								
Other possible symptoms of food allergy		●								
Diet of child (and breast-feeding mother)		●								
EASI		●							●	
Structured allergy history		○								
Skin Prick Test (SPT)		○								
Oral Food Challenge (OFC)		*								
Home dietary trial		*								
EMR notes review										●

V_0 = baseline visit; V_1 = follow-up visit at 24weeks

● All participants; ○ only participants in intervention group; * only participants in intervention group with equivocal structured allergy history/SPT results

POEM: Patient Orientated Eczema Measure; † bother score, itch intensity, parent global assessment; ADQoL: Atopic Dermatitis Quality of Life; IDQoL: Infant Dermatitis Quality of Life; CHU-9D: Children's Health Utility 9D; GAD-7: Generalised Anxiety Disorder 7; EASI: Eczema Area Severity Index

FIGURE

Figure 1: Overview of participant pathway through the study

For peer review only - http://bmjopen.bmj.com/site/about/guidelines.xhtml

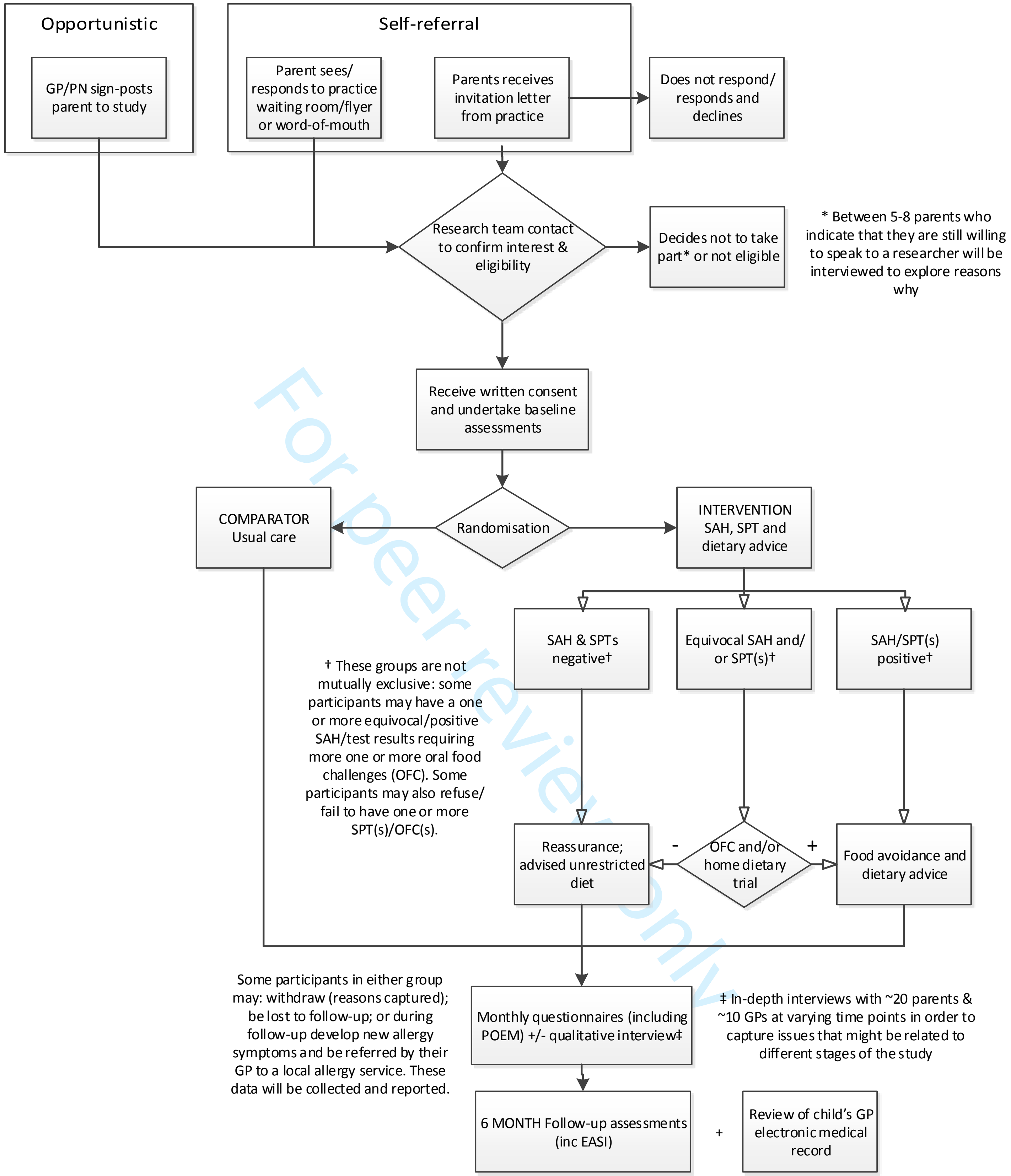

+

For peer review only

Appendix 1: Administrative information

Title

TEST (Trial of Eczema allergy Screening Tests): a single centre, individually randomised, two-group feasibility randomised controlled trial of allergy tests in children with eczema, with economic scoping and nested qualitative study

Trial registration number

ISRCTN: 15397185 (30 July 2018)

World Health Organization Trial Registration Data Set

Data category	Information
Primary registry and trial identifying number	ISRCTN 15397185
Date of registration in primary registry	30 July 2018
Secondary identifying numbers	IRAS: 237046<br>NHS REC: 18/WM/0124
Source(s) of monetary or material support	NIHR School for Primary Care Research
Primary sponsor	University of Bristol
Secondary sponsor(s)	Not applicable
Contact for public queries	Mr Doug Webb, test-study@bristol.ac.uk, 0117 928 7351
Contact for scientific queries	Dr Matthew Ridd FRCGP PhD, m.ridd@bristol.ac.uk, 0117 331 4557
Public title	Trial of Eczema allergy Screening Tests (TEST)
Scientific title	The TEST (Trial of Eczema Allergy Screening Tests) study: feasibility randomised controlled trial with economic scoping and nested qualitative study
Countries of recruitment	England
Health condition(s) or problem(s) studied	Childhood eczema
Intervention(s)	Structured allergy history and skin prick tests; depending on outcome of these tests, reassurance, repeat skin prick test(s), oral food challenge and/or home dietary trial of exclusion or inclusion
Key inclusion and exclusion criteria	Inclusion: aged between 3 months and less than 5 years; eczema diagnosed by an appropriately qualified healthcare professional; mild, moderate or severe eczema (Patient Orientated Eczema Measure (POEM) score>2)<br>Exclusion: medically-diagnosed food allergy or awaiting referral/investigations for possible food allergy; previous investigations for food allergy (does not include home testing)
Study type	Intervention

For peer review only

Date of first enrolment	12 September 2018
Target sample size	80
Recruitment status	Recruiting
Primary outcome(s)	The feasibility of conducting the trial (recruitment, retention, contamination) and collecting the required data: recruitment and retention rates; acceptability of recruitment, intervention and follow-up procedures to parents/carers; acceptability of trial processes and procedures to GPs; development and refinement of a manual on the interpretation of test results and dietary advice to be given; number of participants In the intervention group with positive/negative tests; adherence to dietary advice; contamination of the control group; acceptability and feasibility of collecting clinical outcomes; feasibility and optimise collection of patient-level data on NHS and personal resource use; feasibility of using the CHU-9D in children under 5 years of age; inform eligibility criteria for the future definitive trial; detection bias in the collection of patient-reported outcomes; trial processes and logistics
Key secondary outcomes	Eczema symptoms, measured using POEM; eczema signs, measured using EASI; eczema 'bother' score; itch intensity score; parent global assessment of eczema; other possible symptoms of food allergy; UK diagnostic criteria for atopic dermatitis; main carer anxiety, measured using GAD-7; diet of child and/or mother if child being breastfed by her; adverse events; child and family quality of life, measured using ADQoL, CHU-9D and IDQoL; satisfaction with trial processes, procedures and paperwork; health services utilisation; out-of-pocket expenses/time off work.

Protocol version

Version 2.0 (18 October 2018)

Version		Notes
**Number**	**Date**	
2.0	18.10.18	Section 5.2: addition of missing data collection points (Diet of child and breast-feeding mother at baseline; ADQoL at 8 weeks)<br><br>Section 8.4: change "avoidance of food(s) with dietary advice; and referral via GP for follow-up" to "avoidance of food(s) with dietary advice; and referral to the local NHS allergy services via GP for longer-term follow-up" ; and "Any participants with indeterminate results will be reviewed by an expert allergy panel (co-applicants Boyle & Marriage) …" to "All participants' results will be reviewed by an expert allergy panel (including co-applicants Ridd, Boyle, Marriage and/or Waddell) …"<br><br>Section 9.1: change "Up to 12 GP surgeries" to "At least 12 GP surgeries …";<br><br>Section 9.3 & section 10.2: change "in/at their [own] GP practice" to "at a participating GP practice";<br><br>Section 10.1: description of expression of interest form corrected from "The form will comprise which will comprise POEM, questions asking their opinion of the role of diet/food allergy in their child's eczema, and any

		previous food allergy tests, diagnoses and/or dietary modifications" to "The form asks if they currently have eczema/a medically diagnosed food allergy and the POEM questions"<br><br>Section 11.3: Change from "In addition, we will conduct brief telephone interviews with ~5-8 parents who decline to take part in response to the initial invitation letter or later withdrawal from the trial but indicate that they are willing to discuss reasons why" to "In addition, we will conduct brief telephone interviews with ~5-8 parents who are ineligible, decline to take part or withdrawal from the trial but indicate that they are willing to discuss reasons why"<br><br>Section 16.1: change "Expected SAEs defined in the study protocol (page 39) …" to "Expected SAEs as defined below …"<br><br>Section 18.1: revised project duration/milestones<br><br>Other minor changes (correction of typing errors, changes in research team)
1.0	29.03.18	Submitted/approved by REC/HRA

Funding

NIHR School for Primary Care Research

Contributorship

See main manuscript

Sponsor contact information

Trial sponsor: University of Bristol
Sponsor's reference: 2832
Contact name: Mrs Anna Brooke
Address: Research Enterprise Development, One Cathedral Square, Bristol BS1 5DD
Email: research-governance@bristol.ac.uk
Telephone: 0117 428 4011

Role of study sponsor and funder

The funder and sponsor had no role in the design of this study and will not have any role during its execution, analyses, interpretation of the data, or decision to submit results.

Committees

The Trial Management Group (TMG) comprises all investigators, the trial manager, research and administrative staff, with input from patient/public representatives. Members of the TMG will contribute to the trial in the following ways: trial design and methods; participant recruitment and trial conduct; trial management; trial logistics and cost management; economic evaluation; qualitative study statistical data analysis; and publication. The TMG will meet on a regular basis to oversee the management of the trial. The TMG will be provided with detailed information by the centre staff regarding trial progress. Meetings will be face-to-face with teleconference facilities for TMG members who are unable to be present.

For peer review only

This study was designed and is being delivered in collaboration with the Bristol Randomised Trials Collaboration (BRTC), a UKCRC registered clinical trials unit which, as part of the Bristol Trials Centre, is in receipt of National Institute for Health Research CTU support funding. Members of the BRTC will attend the TMG.

Because this is a low-risk trial, the funder has agreed that the roles of both guiding the Trial Management Group and monitoring trial data will be undertaken by a single Trial Steering/Data Monitoring Committee (TS/DM-C). The TS/DM-C will meet at least three times over the course of the study and comprises four independent members: a chairperson, a biostatistician, a clinician, and a patient representative (parent of child with eczema). Their role will be to provide overall supervision of the trial on behalf of the funder, with a focus on progress of the trial, adherence to the protocol, patient safety and consideration of new information. The committee will review the accruing data and assess whether there are any safety issues that should be brought to the Sponsor's or the participants' attention or any reasons for the trial not to continue. Terms of reference will be drawn up and agreed with members of the TS/DM-C.

For peer review only

The Trial of Eczema allergy Screening Tests (TEST) Study

Parent/Carer Consent Form

Initial box

1. I confirm that I have read and understand the Participant Information Sheet dated 18.10.18 Version 2.0 for the above study. I have had the opportunity to consider the information, ask questions and have had these questions answered satisfactorily. ☐
2. I understand that participation is voluntary and that we are free to withdraw at any time without giving any reason, without my child's medical care or legal rights being affected. ☐
3. I understand that after the study ends, the data collected will be made "open data". I understand that this means the anonymised data will be publicly available and may be used for purposes not related to this study. I understand that it will not be possible to identify me from these data. ☐
4. I understand that relevant sections of my child's medical notes and all information collected for this research may be reviewed by the study team, by the participating NHS Trust to ensure that the research is conducted appropriately. I give permission for these individuals to access my child's records as appropriate. ☐
5. I agree that my child's family doctor (GP) will be told that they are taking part in the study ☐
6. I give consent for the data collected in this trial to be used in future ethically approved studies on the understanding that all information will continue to be kept securely and remain confidential. ☐
7. I give consent to be contacted by a member of the research team with a view to being interviewed about my opinions about allergy testing for eczema and taking part in TEST. I understand that if I am contacted, I will be given more information about the interviews by the research team, I can decide later about taking part and I understand I will be asked to give further consent. ☐
8. I agree for myself and my child to take part in the above-named study. ☐

Name of Participant (Child)	Participant ID	
Name of Parent/Guardian	Signature	Date
Name of person receiving consent	Signature	Date

When completed: 1 (original) for research team, 1 for participant

Funded by NHS National Institute for Health Research

The research was funded by the NIHR Health Technology Assessment (HTA) Programme. The views expressed are those of the author(s) and not necessarily those of the NHS, the NIHR or the Department of Health.

For peer review only - http://bmjopen.bmj.com/site/about/guidelines.xhtml

STANDARD PROTOCOL ITEMS: RECOMMENDATIONS FOR INTERVENTIONAL TRIALS

SPIRIT 2013 Checklist: Recommended items to address in a clinical trial protocol and related documents*

Section/item	ItemNo	Description	Location
**Administrative information**			
Title	1	Descriptive title identifying the study design, population, interventions, and, if applicable, trial acronym	Page 1
Trial registration	2a	Trial identifier and registry name. If not yet registered, name of intended registry	Page 2
	2b	All items from the World Health Organization Trial Registration Data Set	Appendix 1
Protocol version	3	Date and version identifier	Appendix 1
Funding	4	Sources and types of financial, material, and other support	Page 11
Roles and responsibilities	5a	Names, affiliations, and roles of protocol contributors	Page 11
	5b	Name and contact information for the trial sponsor	Page 8/Appendix 1
	5c	Role of study sponsor and funders, if any, in study design; collection, management, analysis, and interpretation of data; writing of the report; and the decision to submit the report for publication, including whether they will have ultimate authority over any of these activities	Page 11/Appendix 1
	5d	Composition, roles, and responsibilities of the coordinating centre, steering committee, endpoint adjudication committee, data management team, and other individuals or groups overseeing the trial, if applicable (see Item 21a for data monitoring committee)	Page 8
**Introduction**			

For peer review only

Background and rationale	6a	Description of research question and justification for undertaking the trial, including summary of relevant studies (published and unpublished) examining benefits and harms for each intervention	Page 3
	6b	Explanation for choice of comparators	Page 3
Objectives	7	Specific objectives or hypotheses	Pages 3-4
Trial design	8	Description of trial design including type of trial (eg, parallel group, crossover, factorial, single group), allocation ratio, and framework (eg, superiority, equivalence, noninferiority, exploratory)	Page 4
**Methods: Participants, interventions, and outcomes**			
Study setting	9	Description of study settings (eg, community clinic, academic hospital) and list of countries where data will be collected. Reference to where list of study sites can be obtained	Page 4
Eligibility criteria	10	Inclusion and exclusion criteria for participants. If applicable, eligibility criteria for study centres and individuals who will perform the interventions (eg, surgeons, psychotherapists)	Page 4/table 2
Interventions	11a	Interventions for each group with sufficient detail to allow replication, including how and when they will be administered	Pages 4-5
	11b	Criteria for discontinuing or modifying allocated interventions for a given trial participant (eg, drug dose change in response to harms, participant request, or improving/worsening disease)	N/A
	11c	Strategies to improve adherence to intervention protocols, and any procedures for monitoring adherence (eg, drug tablet return, laboratory tests)	Page 6
	11d	Relevant concomitant care and interventions that are permitted or prohibited during the trial	Page 5

For peer review only - http://bmjopen.bmj.com/site/about/guidelines.xhtml

Outcomes	12	Primary, secondary, and other outcomes, including the specific measurement variable (eg, systolic blood pressure), analysis metric (eg, change from baseline, final value, time to event), method of aggregation (eg, median, proportion), and time point for each outcome. Explanation of the clinical relevance of chosen efficacy and harm outcomes is strongly recommended	Pages 5-6, table 3
Participant timeline	13	Time schedule of enrolment, interventions (including any run-ins and washouts), assessments, and visits for participants. A schematic diagram is highly recommended (see Figure)	Pages 4 & 6, Figure 1 and Table 3
Sample size	14	Estimated number of participants needed to achieve study objectives and how it was determined, including clinical and statistical assumptions supporting any sample size calculations	Page 6
Recruitment	15	Strategies for achieving adequate participant enrolment to reach target sample size	Pages 4 & 6
**Methods: Assignment of interventions (for controlled trials)**			
Allocation:			
Sequence generation	16a	Method of generating the allocation sequence (eg, computer-generated random numbers), and list of any factors for stratification. To reduce predictability of a random sequence, details of any planned restriction (eg, blocking) should be provided in a separate document that is unavailable to those who enrol participants or assign interventions	Page 4
Allocation concealment mechanism	16b	Mechanism of implementing the allocation sequence (eg, central telephone; sequentially numbered, opaque, sealed envelopes), describing any steps to conceal the sequence until interventions are assigned	Page 4
Implementation	16c	Who will generate the allocation sequence, who will enrol participants, and who will assign participants to interventions	Page 4

For peer review only - http://bmjopen.bmj.com/site/about/guidelines.xhtml

Blinding (masking)	17a	Who will be blinded after assignment to interventions (eg, trial participants, care providers, outcome assessors, data analysts), and how	
	17b	If blinded, circumstances under which unblinding is permissible, and procedure for revealing a participant's allocated intervention during the trial	Page 6
**Methods: Data collection, management, and analysis**			
Data collection methods	18a	Plans for assessment and collection of outcome, baseline, and other trial data, including any related processes to promote data quality (eg, duplicate measurements, training of assessors) and a description of study instruments (eg, questionnaires, laboratory tests) along with their reliability and validity, if known. Reference to where data collection forms can be found, if not in the protocol	Page 6 and Table 3
	18b	Plans to promote participant retention and complete follow-up, including list of any outcome data to be collected for participants who discontinue or deviate from intervention protocols	Page 6
Data management	19	Plans for data entry, coding, security, and storage, including any related processes to promote data quality (eg, double data entry; range checks for data values). Reference to where details of data management procedures can be found, if not in the protocol	Page 6
Statistical methods	20a	Statistical methods for analysing primary and secondary outcomes. Reference to where other details of the statistical analysis plan can be found, if not in the protocol	Page 6
	20b	Methods for any additional analyses (eg, subgroup and adjusted analyses)	N/A
	20c	Definition of analysis population relating to protocol non-adherence (eg, as randomised analysis), and any statistical methods to handle missing data (eg, multiple imputation)	N/A
**Methods: Monitoring**			

For peer review only - http://bmjopen.bmj.com/site/about/guidelines.xhtml

Data monitoring	21a	Composition of data monitoring committee (DMC); summary of its role and reporting structure; statement of whether it is independent from the sponsor and competing interests; and reference to where further details about its charter can be found, if not in the protocol. Alternatively, an explanation of why a DMC is not needed	Page 8
	21b	Description of any interim analyses and stopping guidelines, including who will have access to these interim results and make the final decision to terminate the trial	Page 8
Harms	22	Plans for collecting, assessing, reporting, and managing solicited and spontaneously reported adverse events and other unintended effects of trial interventions or trial conduct	Pages 5-6
Auditing	23	Frequency and procedures for auditing trial conduct, if any, and whether the process will be independent from investigators and the sponsor	Page 8
**Ethics and dissemination**			
Research ethics approval	24	Plans for seeking research ethics committee/institutional review board (REC/IRB) approval	Page 9
Protocol amendments	25	Plans for communicating important protocol modifications (eg, changes to eligibility criteria, outcomes, analyses) to relevant parties (eg, investigators, REC/IRBs, trial participants, trial registries, journals, regulators)	Page 9
Consent or assent	26a	Who will obtain informed consent or assent from potential trial participants or authorised surrogates, and how (see Item 32)	Page 9
	26b	Additional consent provisions for collection and use of participant data and biological specimens in ancillary studies, if applicable	N/A
Confidentiality	27	How personal information about potential and enrolled participants will be collected, shared, and maintained in order to protect confidentiality before, during, and after the trial	Page 9

review only

For peer review only - http://bmjopen.bmj.com/site/about/guidelines.xhtml

Declaration of interests	28	Financial and other competing interests for principal investigators for the overall trial and each study site	Page 11
Access to data	29	Statement of who will have access to the final trial dataset, and disclosure of contractual agreements that limit such access for investigators	Pages 9-10
Ancillary and post-trial care	30	Provisions, if any, for ancillary and post-trial care, and for compensation to those who suffer harm from trial participation	Page 10
Dissemination policy	31a	Plans for investigators and sponsor to communicate trial results to participants, healthcare professionals, the public, and other relevant groups (eg, via publication, reporting in results databases, or other data sharing arrangements), including any publication restrictions	Page 10
	31b	Authorship eligibility guidelines and any intended use of professional writers	N/A
	31c	Plans, if any, for granting public access to the full protocol, participant-level dataset, and statistical code	Page 10
**Appendices**			
Informed consent materials	32	Model consent form and other related documentation given to participants and authorised surrogates	Appendix 2
Biological specimens	33	Plans for collection, laboratory evaluation, and storage of biological specimens for genetic or molecular analysis in the current trial and for future use in ancillary studies, if applicable	N/A

*It is strongly recommended that this checklist be read in conjunction with the SPIRIT 2013 Explanation & Elaboration for important clarification on the items. Amendments to the protocol should be tracked and dated. The SPIRIT checklist is copyrighted by the SPIRIT Group under the Creative Commons "Attribution-NonCommercial-NoDerivs 3.0 Unported" license.

For peer review only
