# Supplementary material for: TEST (Trial of Eczema allergy Screening Tests): protocol for feasibility randomised controlled trial of allergy tests in children with eczema, including economic scoping and nested qualitative study
Source: BMJ Open. 2019 May 9;9(5):e028428. doi: 10.1136/bmjopen-2018-028428 (PMC6527983; doi:10.1136/bmjopen-2018-028428)
Supplement: Reviewer comments [file bmjopen-2018-028428.reviewer_comments.pdf]

PEER REVIEW HISTORY

BMJ Open publishes all reviews undertaken for accepted manuscripts. Reviewers are asked to complete a checklist review form (**http://bmjopen.bmj.com/site/about/resources/checklist.pdf**) and are provided with free text boxes to elaborate on their assessment. These free text comments are reproduced below.

ARTICLE DETAILS

**TITLE (PROVISIONAL)**	TEST (Trial of Eczema allergy Screening Tests): protocol for feasibility randomised controlled trial of allergy tests in children with eczema, including economic scoping and nested qualitative study
**AUTHORS**	Ridd, Matthew; Edwards, Louisa; Santer, Miriam; Chalmers, Joanne; Waddell, Lisa; Marriage, Deborah; Muller, Ingrid; Roberts, Kirsty; Garfield, Kirsty; Coast, Joanna; Selman, Lucy; Clement, Clare; Shaw, Ali; Angier, Elizabeth; Blair, Peter; Turner, Nicholas; Taylor, Jodi; Kai, Joe; Boyle, R

This article was not externally reviewed at BMJ Open. Protocols that have been independently assessed prior to submission to BMJ Open are usually fast-tracked to publication on the grounds that further substantial changes will not be possible. This independent assessment will usually be external, independent review for both a substantial grant award from a non-commercial or government funder and ethics approval.
